# PU-H71 (NSC 750424): a molecular masterpiece that targets HSP90 in cancer and beyond

**DOI:** 10.3389/fphar.2024.1475998

**Published:** 2024-11-05

**Authors:** Sameh Saber, Rasha Abdelhady, Mai A. Elhemely, Elsayed A. Elmorsy, Rabab S. Hamad, Mustafa Ahmed Abdel-Reheim, Attalla F. El-kott, Mohammed A. AlShehri, Kareem Morsy, Ali S. AlSheri, Mahmoud E. Youssef

**Affiliations:** ^1^ Department of Pharmacology, Faculty of Pharmacy, Delta University for Science and Technology, Gamasa, Egypt; ^2^ Pharmacology and Toxicology Department, Faculty of Pharmacy, Fayoum University, Fayoum, Egypt; ^3^ Pharmacology and Toxicology Department, Faculty of Pharmacy, Egyptian Chinese University, Cairo, Egypt; ^4^ School of Medical Sciences, Faculty of Biology, Medicine and Health, The University of Manchester, Manchester, United Kingdom; ^5^ Department of Pharmacology and Therapeutics, College of Medicine, Qassim University, Buraidah, Saudi Arabia; ^6^ Department of Clinical Pharmacology, Faculty of Medicine, Mansoura University, Mansoura, Egypt; ^7^ Biological Sciences Department, College of Science, King Faisal University, Al Ahsa, Saudi Arabia; ^8^ Central Laboratory, Theodor Bilharz Research Institute, Giza, Egypt; ^9^ Department of Pharmaceutical Sciences, College of Pharmacy, Shaqra University, Shaqra, Saudi Arabia; ^10^ Department of Pharmacology and Toxicology, Faculty of Pharmacy, Beni-Suef University, Beni Suef, Egypt; ^11^ Department of Biology, College of Science, King Khalid University, Abha, Saudi Arabia; ^12^ Department of Zoology, Faculty of Science, Damanhour University, Damanhour, Egypt; ^13^ Department of Zoology, Faculty of Science, Cairo University, Cairo, Egypt

**Keywords:** Hsp90, PU-H71 (NSC 750424), tumorigenesis, apoptosis, oncogenic signals

## Abstract

Heat shock protein 90 (HSP90) is a pivotal molecular chaperone with multifaceted roles in cellular health and disease. Herein, we explore how HSP90 orchestrates cellular stress responses, particularly through its partnership with heat shock factor 1 (HSF-1). PU-H71, a selective inhibitor of HSP90, demonstrates significant potential in cancer therapy by targeting a wide array of oncogenic pathways. By inducing the degradation of multiple client proteins, PU-H71 disrupts critical signaling pathways such as MAPK, PI3K/Akt, JAK/STAT, EGFR, and mTOR, which are essential for cancer cell survival, proliferation, and metastasis. We examined its impact on combating triple-negative breast cancer and enhancing the effectiveness of carbon-ion beam therapy, offering new avenues for cancer treatment. Furthermore, the dual inhibition of HSP90A and HSP90B1 by PU-H71 proves highly effective in the context of myeloma, providing fresh hope for patients with this challenging malignancy. We delve into its potential to induce apoptosis in B-cell lymphomas that rely on Bcl6 for survival, highlighting its relevance in the realm of hematologic cancers. Shifting our focus to hepatocellular carcinoma, we explore innovative approaches to chemotherapy. Moreover, the current review elucidates the potential capacity of PU-H71 to suppress glial cell activation paving the way for developing novel therapeutic strategies for neuroinflammatory disorders. Additionally, the present report also suggests the promising role of PU-H71 in JAK2-dependent myeloproliferative neoplasms. Eventually, our report sheds more light on the multiple functions of HSP90 protein as well as the potential therapeutic benefit of its selective inhibitor PU-H71 in the context of an array of diseases, laying the foundations for the development of novel therapeutic approaches that could achieve better treatment outcomes.

## 1 Introduction

**FIGURE 1:**
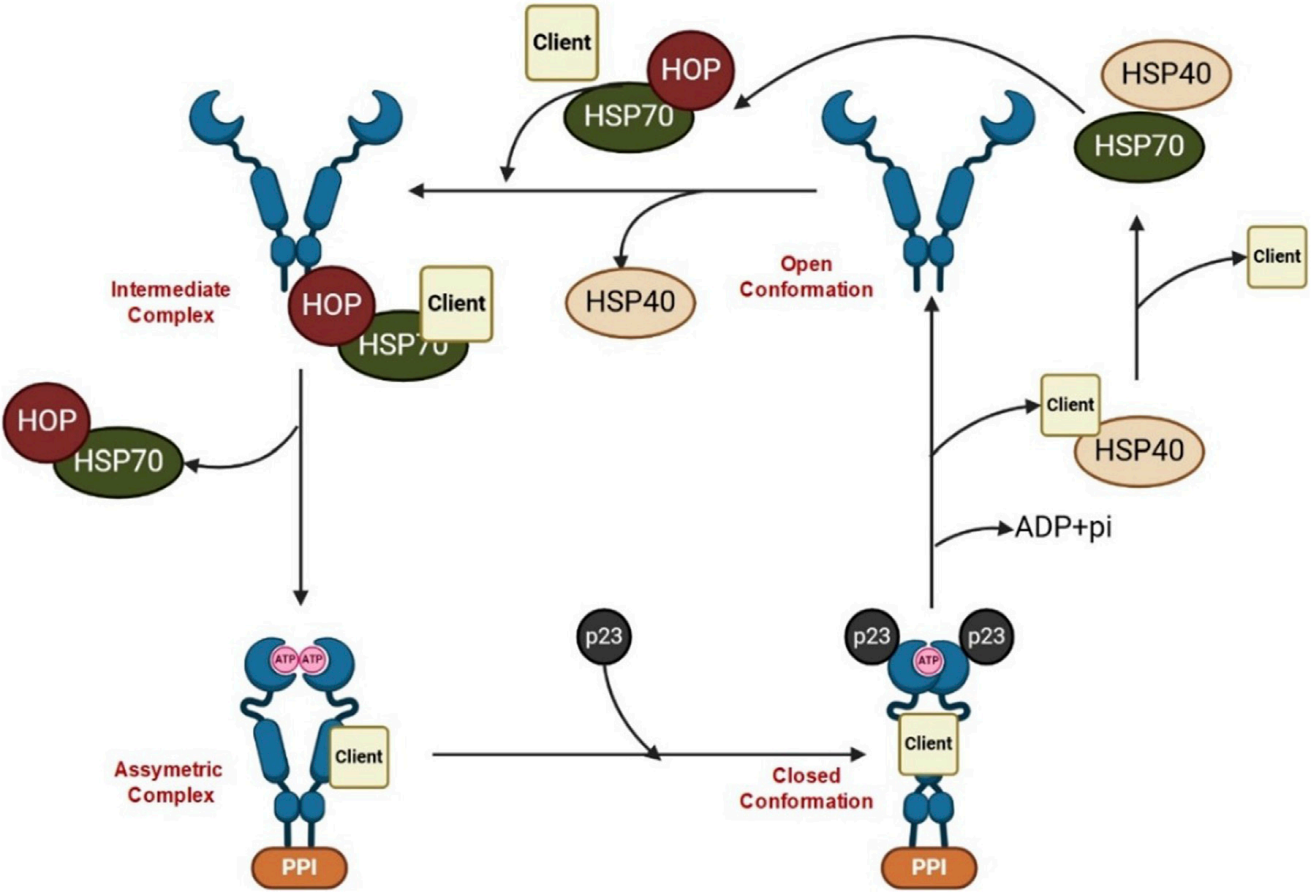
HSP90 cycle.

Heat shock proteins (HSPs) denote a class of highly conserved proteins playing a pivotal role in safeguarding the cells against several forms of stress. Notably, HSPs are central to a range of cellular functions including protein folding, unfolding, assembly, and disassembly. According to their molecular weight, HSPs are classified into main four subgroups, namely, HSP90, HSP70, HSP60, and the small HSP family (Table 1) (Hu et al., 2022). Conspicuously, HSP90 is a leading chaperone protein indispensable for correcting misfolded proteins. On the other hand, HSP70 functions by binding to partially unfolded protein fragments inhibiting their clumping. Meanwhile, HSP60 plays a fundamental role in the proper folding of proteins within specific cellular compartments (e.g., mitochondria). Lastly, the small HSP family, including HSP27, suppresses the aggregation of misfolded proteins (Navarro-Zaragoza et al., 2021). In times of stress, such as exposure to high temperatures, HSPs play a critical role in cell survival by latching onto and stabilizing damaged or partially unfolded proteins until the stressful conditions subside and normal cellular conditions are restored (Kakkar et al., 2014). This allows the proteins to undergo proper folding with the assistance of HSPs. The initial, inactivated state of the HSP90 chaperone cycle is denoted by the closed conformation. Upon binding of ATP, a structural alteration occurs in HSP90, causing it to transition into the open state. In this open state, HSP90 exhibits increased ATPase activity and becomes receptive to client proteins, which can then engage with it for protein folding (Figure 1) (Youssef et al., 2023). HSPs are classified according to their broad structural classes as described in Table 1.

**TABLE 1:** Classification and characteristics of HSPs.

Classification	Subclassification and characteristics
HSP90	HSP90α is primarily found in the cytoplasm
	HSP90β is found in the mitochondria
HSP70	Constitutive HSP70 (HSC70) is found in the cytoplasm
	Inducible HSP70 (HSP72) is produced in response to cellular stress
HSP60	HSP60 (cytosolic)
	HSP60 (mitochondrial)
HSP40	Constitutive HSP40 is found in the cytoplasm
	Inducible HSP40 is produced in response to cellular stress
Small HSPs	HSP27 is involved in the regulation of the actin cytoskeleton
	HSP20 is involved in protection against cellular damage

Inactive Hsp90 (open conformation) forms a dimer at its C terminus, leaving the N termini unassociated, while the Hsp70-captures client proteins and delivers them to Hsp90 with the help of co-chaperones. The co-chaperone Hop binds to both Hsp70 and Hsp90 through its TPR domains, acting as a scaffold to facilitate the transfer of the client protein from Hsp70 to Hsp90 (intermediate complex). Additional co-chaperones, including peptidyl-prolyl cis-trans isomerases (PPIase), associate with Hsp90, leading to the formation of an asymmetric complex. Subsequently, Hsp70 and Hop dissociate, and the co-chaperone p23 replaces them, forming the closed late conformation, where ATP hydrolysis occurs, resulting in the release of the client protein.

Heat shock protein 90 (HSP90) plays a crucial role as a key molecular helper, vital for safeguarding the functional stability and survival of cells when confronted with changing stress factors. Within the purview of HSPs, HSP90 has gained rising attention, fundamentally this could be ascribed to its crucial role in an array of normal physiological cellular processes as well as those correlated with disease progression. Through direct connections, HSP90 was reported to keep proteins associated with cell death in a state that is resistant to programmed cell death (Peng et al., 2022). The wide range of functions exerted by HSP90 is likely attributed to its ability to serve as a helper for numerous client proteins playing indispensable roles in health disorders such as cancer and neurodegenerative diseases (Sumi and Ghosh, 2022).

Targeting and inhibition of HSP90 were achieved by a class of drugs that have been classified as HSP90 inhibitors capable of destabilizing and degrading HSP90 client proteins, with subsequent suppression of multiple oncogenic signaling pathways simultaneously. This multi-target approach suggests that HSP90 inhibitors could be promising therapeutic agents for various neoplasms (Magwenyane et al., 2022). Notably, HSP90 inhibitors, such as geldanamycin derivatives (e.g., 17-AAG and 17-DMAG) and resorcinol-based compounds (e.g., ganetespib), displayed potent anti-cancer activity in preclinical trials and are currently undergoing clinical trials evaluating their safety profiles. The therapeutic benefit of HSP90 inhibitors could potentially exceed anticancer therapy. Markedly, ongoing studies are investigating their potential benefit in neurodegenerative disorders and other health conditions (Luo et al., 2010). HSP90 inhibitors are summarized in Table 2. Table 2 shows the classes of HSP90 inhibitors. The structural diversity of HSP90 inhibitors allows for the development of various drug candidates for cancer and other diseases.

**TABLE 2:** HSP90 inhibitors.

Inhibitor Name	Mechanism of Action	Reference
Geldanamycin	Binds to the N-terminal ATP-binding pocket, inhibiting its chaperone function	Miyata (2005)
17-AAG (Tanespimycin)	Similar mechanism to Geldanamycin but with improved pharmacokinetics	Modi et al. (2011)
17-DMAG	A more soluble derivative of Geldanamycin that inhibits HSP90	Mellatyar et al. (2018)
IPI-504	Inhibits HSP90 by binding to the N-terminal domain	Akram et al. (2018)
NVP-AUY922	Targets the N-terminal ATP-binding site of HSP90	Ueno et al. (2012)
Ganetespib (STA-9090)	Inhibits the chaperone activity of HSP90 by binding to its N-terminal domain	Youssef et al. (2023)
AT13387	Binds to the N-terminal domain, disrupting client protein interactions	Smyth et al. (2012)
PU-H71	Inhibits HSP90 activity by targeting its ATPase function	Trendowski (2015)
Debio0932 (formerly CUDC-305)	Dual inhibitor of both HSP90 and HDAC	Wu et al. (2023)
Pimitespib (TAS-116)	Inhibits the NF-κB signaling pathway by blocking HSP90 activity	El-Kashef et al. (2022)
CNF-2024	Targets the ATP-binding site of HSP90, disrupting its function	Lopes et al. (2007)
SNX-5422	Targets the N-terminal domain but was abandoned due to ocular toxicity issues	Chen et al. (2021)

## 2 Role of HSP90 in tumorigenesis

HSP90 is a crucial molecular chaperone that plays a significant role in cancer biology by facilitating the proper folding, stabilization, and functional regulation of numerous proteins that are essential for tumorigenesis (Birbo et al., 2021). As a prominent member of the heat shock protein family, HSP90 assists in maintaining cellular proteostasis, particularly under the stress conditions often encountered in cancer cells. Its importance in cancer has led to extensive research focusing on HSP90 as a therapeutic target, given its involvement in various oncogenic processes.

HSP90 is known to interact with over 200 client proteins, many of which are involved in critical signaling pathways that control cell growth, survival, and metastasis (Kamal et al., 2004; Xu and Neckers, 2007; Sun et al., 2021). Among these client proteins are receptor tyrosine kinases such as human epidermal growth factor receptor 2 (HER2) and epidermal growth factor (EGFR) (Wang et al., 2016), which are essential for transmitting growth signals from the extracellular environment into the cell interior. HSP90 also interacts with key signaling molecules such as AKT and SRC, which are critical for cell survival and proliferation (Rong and Yang, 2018). HSP90 is also required for the stability and function of transcription factors such as hypoxia inducible factor (HIF-1) and p53, which are responsible for regulating hypoxia responses and controlling the cell cycle, respectively (Albadari et al., 2019; Shirai et al., 2021).

Furthermore, HSP90 stabilizes cell cycle regulators such as CDK4 and CDK6, which are required for cell cycle progression (Sager et al., 2022). In cancer cells, HSP90 protects mutated and overexpressed oncoproteins that would otherwise be degraded. This protective mechanism is especially important because cancer cells frequently experience high levels of cellular stress as a result of rapid proliferation and abnormal protein synthesis. As a result, HSP90 expression levels are typically two to ten times higher in cancer cells than in normal cells (Solárová et al., 2015; Birbo et al., 2021). This overexpression has been linked to a poor prognosis and increased resistance to various therapeutic interventions, highlighting the role of HSP90 in cancer progression.

Hanahan and Weinberg identified several hallmarks of cancer in which HSP90 plays a role. These characteristics include self-sufficiency in growth signals, apoptosis evasion, long-term angiogenesis, and increased tissue invasion and metastasis (Hanahan and Weinberg, 2011). HSP90 allows tumor cells to thrive even in adverse conditions such as nutrient deprivation or therapeutic stress by stabilizing oncogenic proteins involved in these processes (Jaeger and Whitesell, 2019). This ability to support malignant characteristics highlights the potential of targeting HSP90 in cancer therapy as a way to disrupt these critical pathways and induce tumor regression. Understanding the post-translational modifications (PTMs) of HSP90, such as phosphorylation, acetylation, and S-nitrosylation, is critical because they regulate chaperone activity and influence cancer development (Caruso Bavisotto et al., 2020).

## 3 Post-translational modifications of HSP90

### 3.1 Phosphorylation: key modulator of HSP90 function

Phosphorylation is one of the most studied HSP90 PTMs, and it has a significant impact on chaperone activity. Several kinases, such as SRC, BRAF, and casein kinase II (CK2), phosphorylate HSP90, affecting its ability to interact with client proteins (Bachman et al., 2018; Firnau and Brieger, 2022; Zhang et al., 2022). For example, SRC phosphorylates HSP90 at Tyr301, which is required for vascular endothelial growth factor receptor 2 (VEGFR2)-mediated angiogenesis, a critical process in tumor growth (Miyata et al., 2013). HSP90 modulates apoptosome formation via phosphorylation of Ser226 and Ser255, highlighting its role in apoptosis regulation (Mollapour and Neckers, 2012). In leukemia, reduced phosphorylation at these sites strengthens the interaction between HSP90 and APAF1, inhibiting cytochrome c-induced apoptosome assembly and contributing to chemoresistance (Ho et al., 2012).

Client kinases such as WEE1 phosphorylate HSP90, resulting in a feedback loop that improves its chaperoning ability for other kinases such as HER2 and CDK1 (Zhang et al., 2024). This interaction demonstrates the complexities of HSP90’s regulatory mechanisms in cancer. Interestingly, WEE1-mediated phosphorylation can reduce the efficacy of HSP90 inhibitors such as geldanamycin and 17-AAG, posing a challenge for therapeutic strategies. A better understanding of phosphorylation sites may lead to new approaches for sensitizing cancer cells to HSP90 inhibitors and overcoming resistance (Mollapour et al., 2010; Iwai et al., 2012; Lokeshwar, 2012).

### 3.2 Acetylation: impact on client protein stability

Acetylation of HSP90 is another important post-translational modification that can affect its chaperone activity. This modification frequently causes destabilization of client proteins, as seen when histone deacetylase (HDAC) inhibitors or HDAC6 silencing increase HSP90 acetylation levels (Liu P. et al., 2021). Acetylation of several lysine residues on HSP90 impairs its ability to bind client proteins and co-chaperones, including p23, resulting in decreased chaperone efficiency (Woodford et al., 2016; Liu et al., 2023). Importantly, the combination of HDAC inhibitors and HSP90 inhibitors has synergistic effects in certain cancers, particularly leukemia, while antagonistic effects are seen in some solid tumors (Krämer et al., 2014; Chen et al., 2020). These findings emphasize the tumor-specific nature of HSP90 acetylation and the complexities of using combination therapies.

### 3.3 S-nitrosylation: nitric oxide’s role in HSP90 inhibition

S-nitrosylation, or the modification of HSP90 by nitric oxide (NO) at Cys597, adds another layer of regulation by inhibiting the chaperone’s ATPase activity. This PTM impairs HSP90’s ability to stabilize client proteins, especially in endothelial cells where NO-mediated S-nitrosylation inhibits angiogenesis (Siragusa and Fleming, 2016; Rizza and Filomeni, 2020). Interestingly, exogenous NO can inhibit HSP90 in cancer cells, resulting in telomere shortening and reduced telomerase activity, both of which are required for cancer cell immortality (Trepel et al., 2010; Solárová et al., 2015). This finding lends credence to the theory that manipulating NO levels may have therapeutic potential in HSP90-driven cancers.

### 3.4 HSP90’s role in nuclear and chromatin events

Aside from its cytoplasmic functions, HSP90 influences several nuclear events, including the regulation of steroid hormone receptors, such as androgen and estrogen receptors. These receptors, which move between the cytoplasm and the nucleus, are essential in hormone-driven cancers such as breast and prostate cancer (Baker et al., 2018; Kaziales et al., 2020). HSP90 regulates their localization, stability, and transcriptional activity, influencing cancer progression.

HSP90 also interacts with key transcription factors such as heat shock transcription factor 1 (HSF1), which controls the expression of heat shock proteins in response to stress. Inhibition of HSP90 can prolong HSF1 activity, resulting in enhanced heat shock responses, which could be used in cancer therapy (Kijima et al., 2019; Cyran and Zhitkovich, 2022). Furthermore, HSP90 stabilizes the transcriptional repressor BCL6 in diffuse large B-cell lymphoma, HSP90 stabilizes the transcriptional repressor BCL6 in diffuse large B-cell lymphoma, which promotes tumorigenesis by inhibiting tumor suppressor genes such as TP53 (Xu-Monette et al., 2012). Targeting HSP90 in such situations may deactivate these genes and promote cancer cell apoptosis.

HSP90’s involvement in chromatin remodeling expands its role in cancer. The chaperone promotes the activity of chromatin-modifying enzymes, including the histone methyltransferase SMYD3, which is overexpressed in hepatocellular carcinoma and colorectal cancer. HSP90 increases SMYD3 activity, which contributes to the transcription of genes that promote cancer cell proliferation (Abu-Farha et al., 2011; Giakountis et al., 2017). Inhibiting HSP90 in these cancers can reduce SMYD3 activity, providing another therapeutic target.

## 4 HSP90 and heat shock factor 1 (HSF1)

Under stress, the heat shock response (HSR) is stimulated/triggered, leading to the upregulation of various cytoprotective HSPs. HSPs mediate cellular protective effects by alleviating the drastic consequences of aberrantly folded and distorted proteins, thus lessening their detrimental effects on the cell (Collier and Benesch, 2020). In mammals, cellular response to stress could result in destroying cellular proteins and it is principally regulated by the activation of HSF-1. Significantly, HSF-1 could regulate the expression of heat shock genes (Chang et al., 2021). Of note, HSF-1 activation involves multiple steps that start with oligomerization, then nuclear translocation and accumulation, and finally undergo post-translational modifications (Masser et al., 2020). HSP90 is implicated in controlling the activity of HSF. HSP90 forms a binding interaction with HSF-1, maintaining the monomeric form of the transcription factor under normal, non-stressed conditions. When the cell experiences stressors, such as exposure to elevated temperatures (heat shock), or when the function of HSP90 is disrupted, HSF-1 becomes detached from the HSP90 complex (Chin et al., 2023). This release leads to the activation of HSF-1 and its transformation into a trimeric form, and it then moves into the cell nucleus, where HSF-1 initiates an HSR, marked by the synthesis of HSPS such as HSP70 and HSP40 activator (Schmauder et al., 2022).

## 5 PU-H71

**FIGURE 2:**
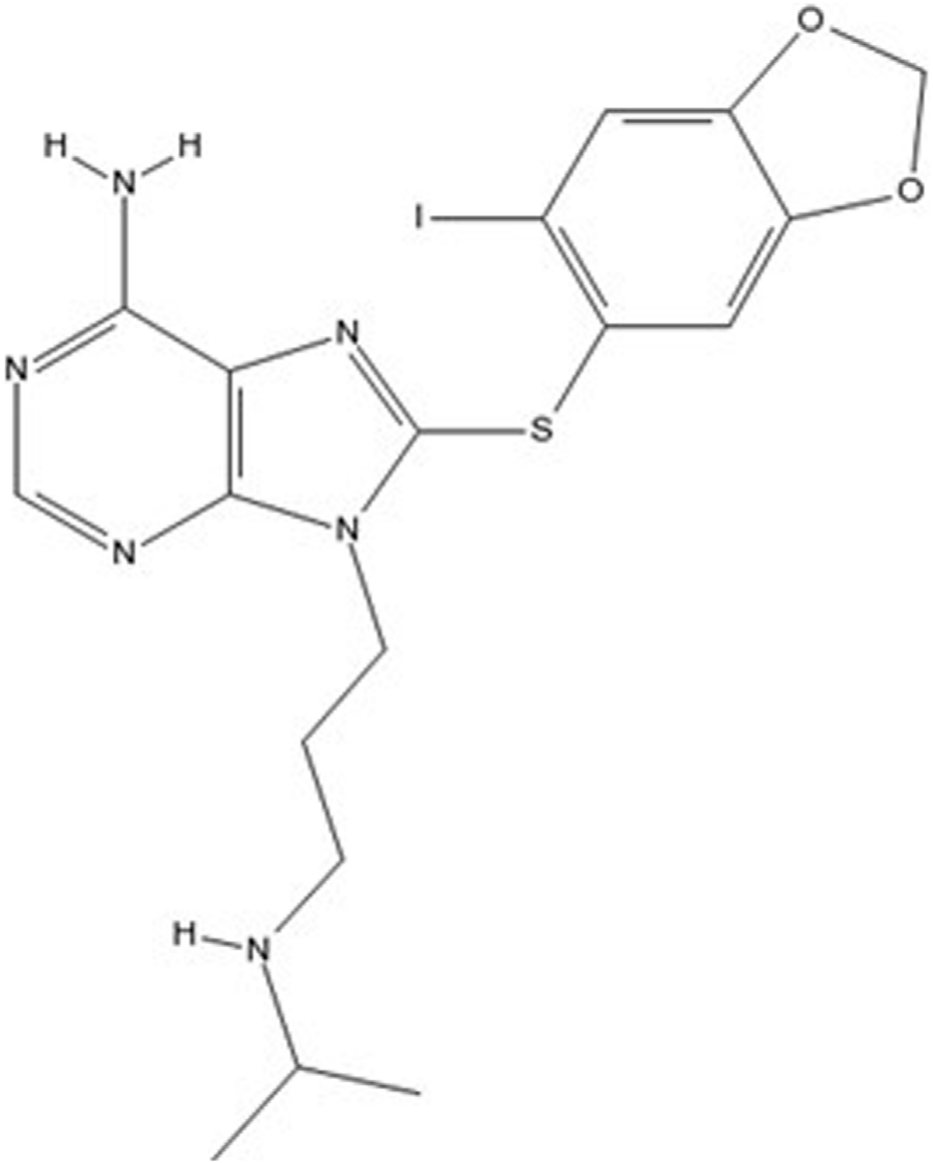
Chemical structure of PU-H71 (8-[(6-iodo-1,3-benzodioxol-5-yl)sulfanyl]-9-[3-(propan-2-ylamino)propyl]purin-6-amine).

PU-H71 (Figure 2) is a potent small-molecule inhibitor that targets HSP90. HSP90 is frequently overexpressed in various cancers, contributing to the stabilization and activity of numerous proteins involved in tumor growth and survival, making it a critical therapeutic target (Mahajan et al., 2024; Rastogi et al., 2024).

PU-H71 binds with high affinity to the ATP-binding site of HSP90, inhibiting its chaperone activity. This binding disrupts the proper folding of client proteins, leading to their ubiquitination and subsequent proteasomal degradation (Schopf et al., 2017). The loss of these client proteins impairs several oncogenic signaling pathways, thereby inhibiting tumor cell proliferation and inducing apoptosis.

Preclinical studies have demonstrated that PU-H71 exhibits significant antitumor activity across a variety of cancer cell lines and xenograft models, particularly those dependent on HSP90 client proteins for growth and survival. These studies have highlighted the ability of PU-H71 to induce tumor regression and prolong survival without causing significant toxicity to normal tissues (Ambati et al., 2014; Trendowski, 2015; Martinet et al., 2022).

Clinical evaluations of PU-H71 have also shown promise. Phase I clinical trials have focused on determining the maximum tolerated dose, pharmacokinetics, and preliminary efficacy of PU-H71 in patients with advanced solid tumors and hematological malignancies. Results from these studies indicate that PU-H71 is well-tolerated and demonstrates antitumor activity, with manageable side effects predominantly involving gastrointestinal disturbances and reversible transaminase elevations. PU-H71 has been included in six clinical studies (NCT03166085; NCT03373877; NCT01581541), of which three (NCT03935555; NCT01393509; NCT01269593) are still active or recruiting, indicating that PU-H71 is the most promising purine-based inhibitor of HSP90 (Gerecitano et al., 2015; Speranza et al., 2018; Pemmaraju et al., 2019).

### 5.1 PU-H71: Discovery

PU-H71 was developed through a rational drug design approach aimed at creating more selective and potent inhibitors of HSP90, compared to previous generations. It is part of a class of compounds synthesized from a purine scaffold, designed to improve both the specificity and solubility of the drug. Earlier compounds like PU3 and PU24FCl, which also targeted HSP90, were optimized through structure-activity relationship studies, refining molecular structures to enhance binding affinity and therapeutic efficacy (Messaoudi et al., 2008). Through this process, PU-H71 emerged as one of the most effective inhibitors, with an IC50 value of approximately 50 nM for HSP90 in epichaperome-containing cells (Ambati et al., 2014).

### 5.2 Pharmacokinetics of PU-H71

The pharmacokinetics of PU-H71 were thoroughly investigated during clinical trials. One of the key findings was the compound’s mean terminal half-life of approximately 8.4 ± 3.6 h, indicating a relatively stable elimination profile across the range of doses tested (Speranza et al., 2018). Biodistribution studies utilizing positron emission tomography imaging revealed that PU-H71 selectively accumulates in tumor tissues that express epichaperomes, with a tumor retention rate observed in about 58% of patients (Dunphy et al., 2020). This tumor-selective accumulation is a promising feature that underscores the drug’s potential as a targeted therapy.

In terms of metabolism, *in vitro* studies revealed that PU-H71 undergoes limited biotransformation, with the primary metabolic pathways involving S-oxidation and O-demethylation (Speranza et al., 2018). Metabolites were present in plasma samples but accounted for less than 10% of the parent drug’s concentration, suggesting that metabolism has a minimal impact on the overall pharmacologic activity of PU-H71 (Speranza et al., 2018).

### 5.3 Development of PU-H71

Preclinical studies have demonstrated that PU-H71 exhibits significant antitumor activity across a variety of cancer cell lines and xenograft models, particularly those dependent on HSP90 client proteins for growth and survival. These studies have highlighted the ability of PU-H71 to induce tumor regression and prolong survival without causing significant toxicity to normal tissues (Ambati et al., 2014; Trendowski, 2015; Martinet et al., 2022).

Following successful preclinical testing, the first-in-human clinical trial of PU-H71 was initiated to evaluate its safety, tolerability, and pharmacokinetic profile. This phase I study involved patients with advanced solid tumors who had not responded to standard therapies. The trial followed a dose-escalation design, with doses ranging from 10 to 470 mg/m^2^, administered intravenously on days 1 and 8 of a 21-day treatment cycle (De Almeida et al., 2020). The results indicated that PU-H71 was well-tolerated at the tested doses, with no dose-limiting toxicities observed. However, some patients experienced grade 2 and 3 adverse events, which were closely monitored. The positive safety profile allowed for the continuation of the drug’s clinical evaluation in subsequent trials. PU-H71 has been included in six clinical studies (NCT03166085; NCT03373877; NCT01581541), of which three (NCT03935555; NCT01393509; NCT01269593) are still active or recruiting, indicating that PU-H71 is the most promising purine-based inhibitor of HSP90 (Gerecitano et al., 2015; Speranza et al., 2018; Pemmaraju et al., 2019).

## 6 Mechanism of action of PU-H71

PU-H71 demonstrates a high degree of selectivity for the ATP-binding sites of HSP90 that are specifically integrated into tumor epichaperome complexes (Dunphy et al., 2020). This selective binding is of paramount importance, as it significantly reduces the potential for off-target effects on healthy tissues where HSP90 does not exist in this altered form (Speranza et al., 2018; Li and Luo, 2023). The unique conformational landscape of HSP90 in tumors allows PU-H71 to engage with its ATP-binding sites effectively (Trendowski, 2015; Tramentozzi and Finotti, 2019). Upon binding, PU-H71 induces critical conformational changes in the HSP90 structure, which stabilizes client protein-HSP90 complexes. This stabilization is not merely a passive interaction; it actively facilitates the recruitment of these complexes to the proteasome, the cellular machinery responsible for degrading misfolded or unneeded proteins (Caldas-Lopes et al., 2009; Li et al., 2018). Consequently, this process leads to the degradation of oncogenic proteins that are essential for tumor cell survival and proliferation. By disrupting the stability of these proteins, PU-H71 effectively undermines the tumor’s ability to maintain its malignant characteristics, thereby presenting a targeted therapeutic strategy against cancer.

The epichaperome represents a highly interconnected network of chaperone proteins that plays a critical role in supporting various cellular processes associated with tumor biology, including proliferation, survival, and invasion (Hadizadeh Esfahani et al., 2018). Within this network, HSP90 acts as a central hub, facilitating the proper folding and function of numerous client proteins that are often dysregulated in cancer. When PU-H71 inhibits HSP90’s activity, it disrupts the integrity of this epichaperome network. The destabilization caused by PU-H71 leads to a significant reduction in the functionality of the epichaperome, impairing its ability to support tumor cell processes. As a result, tumor cells become more susceptible to stressors and less capable of maintaining their survival and proliferative advantages (Speranza et al., 2018; Jhaveri et al., 2020). Ultimately, this disruption contributes to increased tumor cell death, highlighting PU-H71’s potential as an effective therapeutic agent in targeting cancer through the modulation of chaperone networks. By interfering with the epichaperomes’ formation and function, PU-H71 not only targets individual oncogenic proteins but also undermines the broader chaperone-dependent mechanisms that tumors rely on for their growth and resilience.

## 7 Molecular mechanisms of PU-H71

**FIGURE 3:**
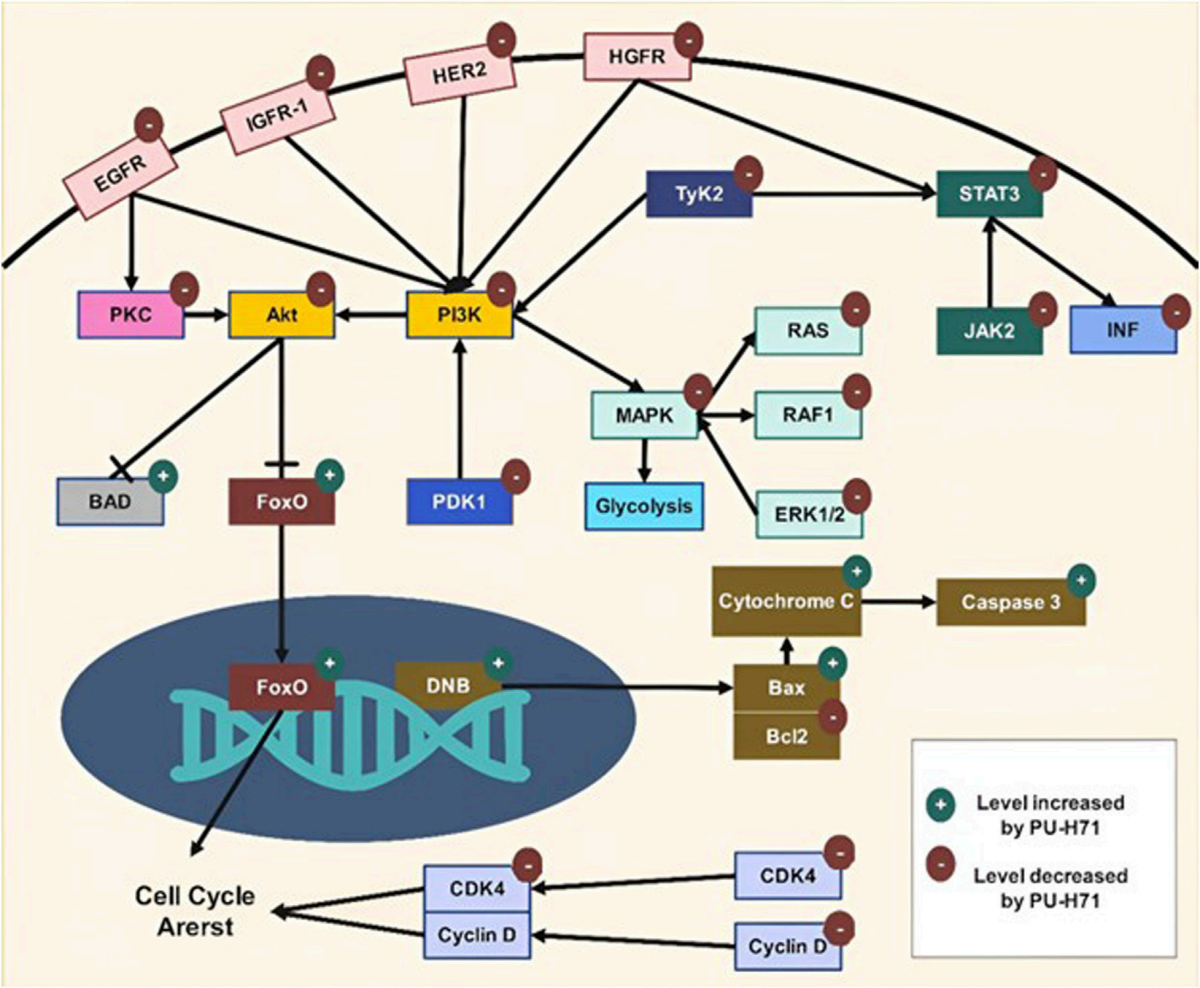
Molecular mechanisms of PU-H71.

Targeting HSP90 by PU-H71 disrupts multiple signaling pathways critical for cancer cell survival, proliferation, and metastasis. The compound exhibits a broad spectrum of anti-cancer activities by inducing the degradation of client proteins involved in key pathways such as Mitogen-Activated Protein Kinase (MAPK), Phosphoinositide 3-Kinase (PI3K)/protein kinase B (Akt), and Janus kinase (JAK)/signal transducers and activators of transcription (STAT) (Figure 3). Additionally, PU-H71 enhances the efficacy of other therapeutic agents and sensitizes cancer cells to treatments like radiation. This multi-faceted approach makes PU-H71 a promising candidate for cancer therapy, addressing the complexity and heterogeneity of tumor biology. Table 3 outlines the various mechanisms through which PU-H71 exerts its anti-cancer effects.

**TABLE 3:** Molecular mechanisms of PU-H71.

Molecular mechanism	Pathways	Effect of PU-H71	Reference
Epichaperomes		PU-H71 binds to Hsp90 within epichaperomes, dismantling them without affecting normal chaperone function The disassembly of epichaperomes restores normal protein-protein interactions, reversing pathological changes in cancer cells	Joshi et al. (2021)
MAPK Signaling	Ras/Raf/MEK/ERK	PU-H71 downregulates Ras, Raf-1, MEK, and ERK, reducing cell proliferation and survival. Disrupts Raf-1 stabilization, inducing degradation of Raf-1 and other oncoproteins. Increases radiosensitivity through p38α MAPK activation	Caldas-Lopes et al. (2009)
	Apoptosis	Inhibits Raf-1, downregulating MAPK pathway, reducing anti-apoptotic factors, enhancing pro-apoptotic factors like Bcl-2 degradation, and leading to increased cell death	Caldas-Lopes et al. (2009)
	Cell Cycle	Induces G2/M phase arrest by downregulating cyclins and CDKs essential for cell cycle progression	Liu et al. (2021a)
Tyrosine kinase 2 (Tyk2)	JAK/STAT, MAPK, PI3K/Akt	Inhibits Tyk2, disrupting cytokine and growth factor signaling, leading to anti-proliferative and pro-apoptotic effects in cancer cells	Caldas-Lopes et al. (2009)
Protein Kinase C (PKC)	PKC Signaling	Induces degradation of PKC by preventing HSP90 chaperoning, reducing PKC-mediated signaling	Yamaki et al. (2011)
ATP	Metabolism	Inhibits Ras/Raf/MAPK pathway, reducing glycolytic ATP production. Decreases PDK1 and Akt activity, reducing oxidative ATP production	Bahar et al. (2023)
DNA double-strand breaks (DSB)	DNA Repair	Sensitizes cancer cells to radiation by inhibiting DSB repair pathways, increasing persistence of γ-H2AX foci, reducing RAD51 foci and DNA-PKcs phosphorylation	Lee et al. (2016), Nickoloff et al. (2017)
Unfolded Protein Response (UPR)	ER Stress	Disrupts HSP90 function, inducing ER stress, activating UPR, leading to apoptosis via ATF4 and CHOP.	Usmani et al. (2010)
Mitochondrial pathway of apoptosis	Apoptosis	Downregulates Bcl-2, upregulates Bax, permeabilizes mitochondrial membrane, releases cytochrome c, activates caspases	Gallerne et al. (2013), Graner et al. (2017)
Endoplasmic reticulum (ER) HSP90	Protein Folding	Could Target gp96 (HSP90B1), destabilizes client proteins, enhancing anti-myeloma drug efficacy	Usmani et al. (2010)
B-cell lymphoma 6 (Bcl6)	Transcription	Inhibits HSP90, destabilizing Bcl6, disrupting its interaction with co-repressors, leading to reduced proliferation and survival of Bcl6-dependent lymphoma cells	Cerchietti et al. (2009)
Hepatocyte growth factor receptor (HGFR)	c-Met Signaling	Inhibits HSP90, leading to c-Met degradation, reducing tumor growth	Breinig et al. (2009)
Protein kinase B (Akt)	PI3K/Akt Signaling	Inhibits HSP90, leading to Akt degradation, reducing phosphorylation at Thr308 and Ser473, disrupting survival and proliferation pathways	Giulino-Roth et al. (2017)
Insulin-like growth factor receptor 1 (IGF1R)	PI3K/Akt, MAPK	Inhibits IGF1R, reducing downstream signaling, promoting apoptosis, and inhibiting cell proliferation	Ambati et al. (2014)
Cyclin Dependent Kinase 4 (CDK4)	Cell Cycle	Downregulates CDK4, cyclin D1, and phosphorylated Rb, inhibiting G1 to S phase transition, causing cell cycle arrest	Knudsen and Witkiewicz (2017)
Interferon (INF)	Inflammatory Response	Reduces IFNγ release, inhibiting activation of inflammatory pathways	Lisi et al. (2013)
Janus kinase 2/signal transducers and activators of transcription 3 (JAK2/STAT3)	JAK/STAT Signaling	Inhibits HSP90, leading to degradation of JAK2 and decreased phosphorylation of STAT3 and STAT5, inhibiting their transcriptional activity	Jego et al. (2019) Niu et al. (2021)
Human Epidermal Growth Factor Receptor 2 (HER2)	Receptor Tyrosine Kinase	Inhibits HSP90, leading to HER2 degradation, reducing activation of downstream signaling pathways like PI3K/Akt and MAPK, promoting apoptosis and reducing cell proliferation	Kale et al. (2020)
Epidermal Growth Factor Receptor (EGFR)	Receptor Tyrosine Kinase	Inhibits HSP90, leading to degradation of EGFR, reducing its activation and downstream signaling pathways like PI3K/Akt and MAPK, inhibiting cell proliferation and survival	Martinelli et al. (2017), Salama et al. (2019), Guerra et al. (2021)
Mammalian Target of Rapamycin (mTOR)	PI3K/Akt/mTOR	Inhibits HSP90, leading to degradation of mTOR, disrupting mTORC1 and mTORC2 signaling, reducing cell growth, proliferation, and survival	Giulino-Roth et al. (2017)

### 7.1 Epichaperomes

Epichaperomes are novel type of molecular structures formed by the assembly of chaperones and co-chaperones, representing a significant advance in our understanding of cellular stress responses, particularly in cancer and neurodegenerative disorders (Rodina et al., 2016). Unlike traditional chaperones, which help with protein folding and aggregation temporarily, epichaperomes form stable oligomeric complexes that remain in cells, fundamentally altering cellular proteostasis (Joshi et al., 2018; Yan et al., 2020; Ginsberg et al., 2021). These long-lived structures are critical in modulating protein-protein interactions (PPIs) within cells, especially under chronic stress conditions (Rodina et al., 2023; Chakrabarty et al., 2024). The formation of epichaperomes in diseased tissues provides new insights into disease mechanisms as well as potential therapeutic avenues, making them a promising intervention target.

Epichaperomes are derived from the chaperome, a network of over 300 chaperones and associated factors that regulate protein folding and quality control under normal conditions (Inda et al., 2020). However, epichaperomes differ from conventional chaperones in terms of stability and function. They serve as scaffolding platforms, reorganizing PPIs and altering cellular function in response to chronic stress, particularly in cancer cells (Ginsberg et al., 2023). Their specificity for diseased cells and tissues, as opposed to the widespread presence of traditional chaperones in healthy cells, makes them an appealing therapeutic target. In cancer, epichaperomes promote cell survival by supporting hyperconnected PPI networks, allowing malignant cells to survive in hostile microenvironments and evade therapeutic treatments (Digwal et al., 2022). Tumors with high epichaperomes expression often exhibit heightened vulnerability to specific inhibitors that disrupt these pathological structures.

A key therapeutic discovery is that small molecules, such as PU-H71, can selectively target epichaperomes and dismantle them without interfering with normal chaperone activity (Jhaveri et al., 2020). PU-H71 binds to Hsp90 within the epichaperome and induces conformational changes that cause the epichaperome to disassemble into its constituent chaperones. The disassembly of epichaperomes restores normal interactome structures, reversing pathological PPI changes. This therapeutic strategy has shown particular promise in cancers like Ras-driven pancreatic cancer, which has redundant signaling pathways that contribute to drug resistance (Joshi et al., 2021). In these cases, epichaperomes maintain interactome hyperconnectivity, which occurs when cancer cells rely on highly connected networks of PPIs to survive. Approximately 50%–60% of tumors express epichaperomes, with those displaying the highest levels of epichaperomes expression being characterized by extreme interactome hyperconnectivity and increased vulnerability to therapeutic interventions (Merugu et al., 2020). Furthermore, the breakdown of the epichaperome causes significant changes in cellular signaling pathways involved in survival and proliferation. For instance, decreased phosphorylation of key signaling proteins such as phospho-SYK and phospho-STAT5 has been documented, highlighting the downstream effects of epichaperome disruption on oncogenic signaling cascades (Roboz et al., 2018).

This approach exploits the vulnerability in cancer cells by pharmacologically manipulating epichaperome-mediated protein-protein interaction (PPI) networks. By forcing the interactome to reach maximum hyperconnectivity, cancer cells become more sensitive to existing therapies (Joshi et al., 2021). This strategy reactivates pre-existing protein pathways, preventing cancer cells from utilizing alternative signaling mechanisms to evade treatment. Unlike traditional therapies that target specific genetic mutations or protein aberrations, this method aims to control the entire interactome, thereby shutting down compensatory pathways that contribute to drug resistance (Rodina et al., 2016; Carter et al., 2023). Experiments in pancreatic cancer models, including cell lines, patient biospecimens, and xenografts, show that pharmacologically induced hyperconnectivity significantly improves the efficacy of current treatments (Joshi et al., 2021). By priming cancer cells with PU-H71 to induce a hyperconnected state, the efficacy of previously ineffective therapies was increased. This novel treatment paradigm represents a promising approach to overcoming drug resistance in cancers marked by high epichaperome expression and interactome plasticity (Joshi et al., 2021). PU-H71, by inducing hyperconnectivity, targets entire PPI networks rather than individual molecular nodes, resulting in a more comprehensive and effective therapeutic strategy.

In clinical settings, PU-H71 has shown promising results, especially in cancers with high epichaperome levels. Preclinical and early clinical trials have shown that patients with acute myeloid leukemia, which is characterized by elevated epichaperome expression, respond well to PU-H71 treatment, with some achieving long-term remission (Li and Adams, 2023; Seipel et al., 2023). These findings highlight the potential of PU-H71 as a therapeutic agent in cancers where the epichaperome plays an important role in disease progression.

### 7.2 Mitogen-activated protein kinase kinase (MAPK) signaling

PU-H71 treatment leads to a potent reduction in the proliferative, anti-apoptotic, and invasive potential of tumors. Among the pathways affected, PU-H71 induces downregulation of components of the Ras/Raf/MAPK pathway, contributing to its anti-proliferative effects (Pastena et al., 2024). In chronic myelogenous leukemia cells, PU-H71 was shown to identify the aberrant signalosome, with a focus on the Raf-MAPK pathway (Moulick et al., 2011). By inhibiting HSP90, PU-H71 destabilizes oncoproteins associated with the MAPK pathway, leading to their downregulation and impairment of downstream signaling (Seipel et al., 2023). PU-H71 has also been found to increase the radiosensitivity of breast cancer cells metastasized to visceral organs. The radiosensitizing effect of PU-H71 in combination with radiation therapy correlates with increased activation of p38α MAPK (García-Flores et al., 2023). Remarkably, recent studies have identified p38α MAPK as a potential tumor suppressor owing to its capacity to interfere with malignant cell transformation *via* regulating tissue homeostasis. This effect could be ascribed to modulating signals that balance cell proliferation and differentiation or induce apoptosis. As previously noted, PU-H71-mediated inhibition of HSP90 could improve the response of tumor cells to radiotherapy through modulation of p38 MAPK signaling.

Additionally, PU-H71 was reported to target MAPK pathways thus consequently decreasing tumor cell resistance to other anti-cancer treatment regimens enhancing their efficacy. Furthermore, combined administration of PU-H71 and the anti-cancer prodigiosin, displayed remarkable synergistic effects against malignant cells, that could be ascribed to targeting different aberrantly regulated pathways including MAPK (Anwar et al., 2020).

Moreover, PU-H71 was documented to disrupt the stabilization of v-Raf-1 (Raf-1) and other oncogenic proteins, leading to their degradation (Trendowski, 2015). It is noteworthy to highlight that, Raf-1 is a critical component of the MAPK signaling cascade aberrantly activated in cancer cells (Beeram et al., 2005).

Besides, PU-H71 anticancer properties could be due to the reported downregulation of the components of the Ras/Raf/MAPK pathway. The activation of such a pathway is initiated by Ras (a small GTPase) phosphorylation and activation by growth factor receptors. Subsequently, activated Ras interacts with Raf-1, leading to activation of the latter (Terrell and Morrison, 2019; Nolan et al., 2021; Ullah et al., 2022). Herein, the pathway activation could be disrupted by PU-H71 treatment through hampering Ras-Raf-1 interaction, thereby inhibiting Raf-1 activation and consequent downstream signaling through MEK and ERK, which are essential for cell proliferation and survival (Caldas-Lopes et al., 2009; Ambati et al., 2014).

Intriguingly, PU-H71 was reported to induce apoptosis in cancer cells that could be also ascribed to the observed Raf-1 inhibition. Also, MAPK pathway suppression following PU-H71 treatment resulted in a significant downregulation of anti-apoptotic factors that triggered the degradation of pro-survival proteins like B-cell lymphoma-2 (Bcl-2) (Gallerne et al., 2013). The restoration of balance between pro-apoptotic and anti-apoptotic signals leads to increased malignant cell death (Yamaki et al., 2011).

Anti-cancer effects of PU-H71 could result from its capacity to induce G2/M phase arrest interfering with cell cycle progression. This could be linked to the reported downregulation of cyclins and cyclin-dependent kinases (CDKs) that are necessary for cell cycle progression (Liu H. et al., 2021). In addition, Raf-1 inhibition with subsequent suppression of the MAPK pathway could probably contribute to the reported cell cycle arrest (Bahar et al., 2023).

### 7.3 Tyrosine kinase 2 (Tyk2)

Tyk2 belongs to the Janus kinase (JAK) family of tyrosine kinases, which are closely implicated in cytokine and growth factor signaling pathways playing an essential for cancer progression (Wöss et al., 2019). Tyk2 was reported to be aberrantly activated in different types of neoplasms (e.g., acute myeloid leukemia, and solid tumors including breast cancer) (Wöss et al., 2019). Aberrant activation of Tyk2 was documented to facilitate a range of abnormal characteristics of cancer cells including abnormal cellular growth and proliferation alongside invasion. Fundamentally, abnormal Tyk2 activation results in the activation of downstream signaling cascades, among these the signal transducers and activators of transcription (STAT), MAPK, and Phosphoinositide 3-kinase (PI3K)/Protein Kinase B (Akt) pathways were reported to be the most important. Meanwhile, suppression Tyk2 cascade was shown to interrupt such oncogenic signaling cascades, which could potentially contribute to its anti-tumor effects in preclinical cancer models (Caldas-Lopes et al., 2009; Kucine et al., 2015).

Remarkably, HSP90 is pivotal for Tyk2 stability and function (Akahane et al., 2016; Reynolds and Blagg, 2024), where HSP90 suppression following PU-H71 treatment has been reported to cause Tyk2 and inactivation of Tyk2, in various cancer models. In an earlier investigation on triple-negative breast cancer (TNBC) cells, PU-H71 exposure caused a remarkable downregulation of multiple oncogenic proteins, including components of the Ras/Raf/MAPK pathway. This could lead to further downregulation of Tyk2 (Ravandi et al., 2003; Dimri and Satyanarayana, 2020).

### 7.4 Protein kinase C (PKC)

HSP90 is important for the stability and function of many protein kinases like PKC (Kawano et al., 2021). PU-H71 binds to HSP90 and prevents it from chaperoning and stabilizing client proteins like PKC (Yamaki et al., 2011). Without the support of HSP90, PKC becomes misfolded and targeted for proteasomal degradation. This leads to a reduction in cellular PKC levels and inhibition of PKC-mediated signaling. The PKC family consists of 10 isozymes that regulate diverse cellular processes (Kawano et al., 2021). By inhibiting HSP90, PU-H71 is expected to affect multiple PKC isoforms simultaneously. However, the relative potency of PU-H71 against each PKC isoform has not been reported.

PU-H71 has been shown to downregulate components of the MAPK signaling pathway, which includes PKC. Specifically, PU-H71 induces the degradation of activated Akt, a key PKC substrate (Yamaki et al., 2011). The documented suppression of PKC signaling has markedly contributed to reported PU-H71 antitumor effects against TNBC. In addition to the reported effects on PKC, PU-H71 also has demonstrated suppression of activated glial cells in experimental autoimmune encephalomyelitis (EAE) and an animal model of multiple sclerosis (Lisi et al., 2013). Additionally, PU-H71’s anti-inflammatory properties could be mediated through the inhibition of PKC and other inflammatory signaling pathways.

### 7.5 Adenosin triphosphate (ATP)

Interestingly, PU-H71-mediated downregulation of Ras/Raf/MAPK pathway components was shown to (Bahar et al., 2023) decrease the rate of glycolytic ATP generation and consequently be implicated in regulating glycolysis and ATP production. Besides, HSP90 was reported be principally involved in regulating the 3-phosphoinositide-dependent protein kinase-1 (PDK1)-Akt signaling pathway (Basso et al., 2002; Xue et al., 2014; Bao et al., 2021). Markedly, HSP90 inhibition by PU-H71 could trigger PDK1 degradation, subsequently suppressing the Akt pathway that would then lead to a profound decline in both the activity of mitochondrial metabolism and oxidative ATP production (Mookerjee et al., 2017).

Moreover, PU-H71 was reported to promote apoptosis as well as degradation of oncogenic proteins that drive both uncontrolled cell proliferation (Speranza et al., 2018) this effect could be mainly mediated by reducing the demand for ATP by these cellular processes, plus decreasing overall ATP utilization.

### 7.6 DNA double-strand breaks (DSB)

Particularly, PU-H71 has a significant capability of sensitizing malignant cells to heavy ion radiation that could be owing to the observed inhibition of DSB repair pathways (Lee et al., 2016) as well as increasing the persistence of γ-H2AX foci that represent markers of unrepaired DSBs, in irradiated tumor cells (Lee et al., 2016). This finding suggests PU-H71-induced impairment of radiation-induced DSB repair mechanisms. Also, the displayed sensitization of malignant cells to radiation was attributed to the decreased formation of RAD51 foci as well as diminished phosphorylation of DNA-PKcs in irradiated tumor cells. Reportedly, both RAD51 and DNA-PKcs are DNA repair proteins central to homologous recombination and non-homologous end-joining DSB repair pathways, respectively. Moreover, RAD51 was reported to be upregulated in several neoplasms and it was responsible for chemoresistance, poor cancer prognosis, as well a higher incidence of metastasis (Nickoloff et al., 2017). Significantly, PU-H71 was shown to improve the efficacy of radiotherapy *via* suppressing radiation-induced DSB repair in irradiated tumor cells (Lee et al., 2016) eventually leading to increased cell death.

PU-H71-induced restraining of DSB repair could potentially be implicated in the observed sensitization of various cancer cell lines to heavy ion radiation, including, human lung cancer cells (Lee et al., 2016). As previously reported, PU-H71 promoted the cytotoxic properties of heavy ion radiation as reflected by both reduced clonogenic survival and increased cell death. Interestingly, the reported radio-sensitizing actions of PU-H71 were explicit to tumor cells and did not affect the survival of normal human fibroblasts (Li et al., 2016). Such precise sensitization, recommends PU-H71 as a novel therapeutic agent for enhancing the efficacy of heavy ion radiotherapy.

### 7.7 Unfolded protein response (UPR)

PU-H71 displayed complex and multiple effects on the UPR including several mechanisms eventually promoting malignant cell apoptosis. Firstly, it was noted that PU-H71 could generate Endoplasmic reticulum (ER) stress probably by suppressing HSP90 normal function. The documented inhibition of HSP90 function accelerates the compilation of both unfolded or misfolded proteins in the ER, triggering the UPR (Gallerne et al., 2013). Of note, UPR represents a cellular response to ER stress that objectively restores protein homeostasis through upregulating certain chaperones including Glucose-regulated protein 78 (GRP78), GRP94, and calreticulin. Outstandingly, PU-H71 triggers the UPR through the induction of the splicing of X-box binding protein 1 (XBP1) mRNA, defined as a key marker of UPR activation (Gallerne et al., 2013).

The main aims of UPR include restoration of protein homeostasis or induction of apoptosis if the stress persists. Apoptosis induction is potentially through activating the downstream transcription factors such as transcription factor 4 (ATF4) and C/EBP homologous protein (CHOP) promoting the expression of pro-apoptotic genes (Rah et al., 2016; Hu et al., 2019).

### 7.8 Mitochondrial pathway of apoptosis

One of the major actions of PU-H71 is the activation of the mitochondrial pathway of apoptosis *via* upregulation of pro-apoptotic proteins (e.g., Bcl-2-Associated X Protein (Bax)) alongside downregulation of anti-apoptotic proteins (Bcl-2) followed by enhanced permeabilization of the mitochondrial membrane with subsequent release of cytochrome c and caspases activation (Gallerne et al., 2013; Graner et al., 2017).

Mechanistically, the capacity of PU-H71 to trigger apoptosis was underlined by activating this mitochondrial pathway in multiple cancer cell lines such as melanoma, cervix, colon, liver, and lung cancer cells (Rastogi et al., 2024). Interestingly, previous findings displayed that PU-H71-induced apoptosis could potentially attenuate malignant cell resistance attributed to overexpression of the anti-apoptotic protein Bcl-2 (Gallerne et al., 2013). Previous findings also highlighted the remarkable contribution of the pro-apoptotic protein Bax to PU-H71-induced apoptosis where Bax-deficient cells were shown to resist PU-H71-elicited apoptosis (Kubra et al., 2020). Conversely, such resistance was partially overcome by combined administration of PU-H71 treatment with other anticancer therapies (e.g., cisplatin or melphalan) (Gallerne et al., 2013). In addition to apoptosis induction, PU-H71 was shown to cause cell cycle arrest at the G2-M phase that could be mediated through cyclin D1 downregulation (Ambati et al., 2014).

### 7.9 Endoplasmic reticulum (ER) HSP90

Over the past decade, gp96 or HSP90B1 also referred to as grp94, the ER HSP90 family member, has attracted distinct attention. This attention was due to gp96’s remarkable role in chaperoning antigenic peptides, enabling their presentation in the antigen-cross-presentation pathway (Marzec et al., 2012; Duan et al., 2021). Outstandingly, immunizations utilizing pure tumor-derived gp96-peptide complexes have displayed promising preclinical and clinical results in the treatment of multiple myeloma (Randazzo et al., 2012). *In-vitro* investigations demonstrated that bortezomib (disturbing protein anti-myeloma drug), rendered gp96 knockdown cells more susceptible. In addition, gp96 knockdown cells demonstrated a remarkable sensitivity to thalidomide alongside increased cell death induced by genotoxic drugs like melphalan and doxorubicin. These outcomes suggest that gp96 could potentially be involved in diminishing the efficacy of anti-myeloma therapies, meanwhile, gp96 knockdown augmented myeloma cells’ response to those agents (Usmani et al., 2010). Nevertheless, gp96 knockdown negatively affected the anti-myeloma activity of PU-H71 suggesting that the acute cytotoxic effect of HSP90 inhibitors is mediated through modulating both cytosolic HSP90 in addition to gp96 in the ER. PU-H71 displayed *in vitro* anti-myeloma efficacy as well. These findings highlight that HSP90 inhibitors, including PU-H71, target both cytosolic HSP90 and the ER-resident HSP gp96 to exert their anti-myeloma effects.

### 7.10 B-cell lymphoma 6 (Bcl6)

BCL6 is identified as a master transcription factor regulating T follicular helper cell proliferation, which is central to germinal center development and B cell proliferation. Remarkably, mutations in BCL6 could result in the development of B cell lymphomas that could be owing to unchecked B cell growth, therefor such mutations could be used as diagnostic markers for B cell lymphomas and other cancers (Mintz and Cyster, 2020).

Interestingly, HSP90 plays a pivotal role in the stability as well as the function of the transcription factor BCL6 (Hoter et al., 2018) Herein, PU-H71 binding to the N-terminal ATP/ADP pocket of HSP90 leads to a prominent inhibition of its ATPase activity plus a profound disruption of HSP90 chaperone function with consequent BCL6 destabilization and degradation (Sidera and Patsavoudi, 2014), reducing its activity. This effect was reflected by the reported decline in luciferase activity of BCL6 target promoters in cell lines following exposure to PU-H71.

The downregulation of BCL6 by PU-H71 is thought to be mediated through multiple mechanisms including, reduced stability and increased degradation of the BCL6 protein (Sidera and Patsavoudi, 2014); disruption of the interaction between BCL6 and its transcriptional co-repressors, such as Silencing Mediator of Retinoid and Thyroid hormone receptors (SMRT), Nuclear receptor corepressor (N-CoR), and BCL6 corepressor (BCoR) (Briones, 2010; Kuai et al., 2018; McLachlan et al., 2022); Interference with the autoregulatory loop that maintains high BCL6 expression in lymphoma cells (Yang and Green, 2019); and derepression of BCL6 target genes involved in cell cycle control, DNA repair, metabolism, and immune regulation. This results in the suppression of the proliferation and survival of BCL6-dependent lymphoma cells, making PU-H71 a promising therapeutic agent for BCL6-driven lymphomas.

### 7.11 Hepatocyte growth factor receptor (HGFR)

The hepatocyte growth factor receptor (HGFR), also known as c-Met, is a receptor tyrosine kinase that is primarily expressed on epithelial cells. Its only known high-affinity ligand is hepatocyte growth factor (HGF) (Fu et al., 2021). The binding of HGF to c-Met activates the receptor, leading to tyrosine phosphorylation and activation of downstream signaling pathways such as MAPK, STAT, and PI3K/Akt (Zhang et al., 2018). Deregulated c-Met signaling, due to factors like receptor overexpression, ligand overexpression, or activating mutations, is implicated in the development, progression, and metastasis of many human cancers. Therefore, c-Met represents an attractive target for anti-cancer therapies, and various agents targeting the HGF/c-Met pathway are under development, including small molecule inhibitors, antibodies, and decoy receptors (Matsumoto et al., 2017).

PU-H71 was found to potently increase the degradation of c-Met as a client protein to HSP90. Treatment with PU-H71 resulted in reduced c-Met protein levels and decreased phosphorylation/activation of c-Met in various cancer cell lines. The inhibition of c-Met by PU-H71 was associated with reduced tumor growth in preclinical cancer models, without causing significant toxicity (Speranza et al., 2018). The mechanism by which PU-H71 inhibits c-Met is through its ability to bind and inhibit the HSP90 chaperone. By disrupting the HSP90-c-Met interaction, PU-H71 leads to the destabilization and degradation of the c-Met receptor, thereby suppressing its oncogenic signaling (Giulino-Roth et al., 2017). This indirect targeting of c-Met via HSP90 inhibition represents a promising therapeutic approach for cancers driven by deregulated c-Met activity.

### 7.12 Protein kinase B (Akt)

HSP90 was found to have an important role in the PDK1-Akt signaling pathway through binding to PDK1. Meanwhile, proteasome-dependent degradation of PDK1 was demonstrated following HSP90 inhibition by PU-H71 (Yamaki et al., 2011). Akt is a client protein of HSP90 and requires HSP90 for its stability and activation. PU-H71 binds to the N-terminal ATP/ADP pocket of HSP90, inhibiting its ATPase activity. This leads to the destabilization and degradation of Akt by the proteasome (Giulino-Roth et al., 2017). Akt activation requires phosphorylation at two regulatory sites: Thr308 in the activation segment by PDK1 and Ser473 in the hydrophobic motif by mTORC2 (Baffi et al., 2021). The binding of PU-H71 to HSP90 inhibits the phosphorylation of Akt at these sites, preventing its activation (Giulino-Roth et al., 2017). The degradation of PDK1 by PU-H71 also reduces the phosphorylation of Akt at Thr308.

Active, phosphorylated Akt regulates a panel of proteins that control proliferation, growth, survival, and metabolism (Hoxhaj and Manning, 2020). Inhibition of Akt by PU-H71 disrupts these downstream pathways. For example, Akt phosphorylates and inactivates the Bcl-2-associated death promoter (proapoptotic protein Bad) (Trendowski, 2015; Seipel et al., 2023). Degradation of Akt by PU-H71 leads to dephosphorylation of Bad, allowing it to induce apoptosis. Additionally, Akt phosphorylates the forkhead box O (FoxO) transcription factors, inducing their nuclear exclusion and inhibition (Orea-Soufi et al., 2022). This effect was restrained by PU-H71 suppressing Akt activity, leading to nuclear localization of FoxO with subsequent upregulation of genes involved in cell cycle arrest and apoptosis.

### 7.13 Insulin-like growth factor receptor 1 (IGF1R)

Insulin-like growth factor receptor 1 (IGF1R), represents a receptor tyrosine kinase crucial for mediating the effects of insulin-like growth factor 1 (IGF-1) (Ambati et al., 2014). Upon binding to IGF1R, IGF-1 activates the receptor’s intrinsic kinase activity, leading to autophosphorylation and activation of downstream signaling cascades essential for promoting cell growth, proliferation, and survival, including PI3K/Akt and Ras/Raf/MEK/ERK (Tahimic et al., 2013).

Significantly, PU-H71 was reported to specifically target IGF1R inhibiting its phosphorylation and then consequently suppressing downstream signaling pathways that eventually led to decreased proliferation and apoptosis induction in malignant cells. Earlier investigations reported that PU-H71 could effectively decrease the viability of various cancer cell lines overexpressing IGF1R (Breinig et al., 2011; Terwisscha Van Scheltinga et al., 2014; Trendowski, 2015). Remarkably, suppression of IGF1R signaling following PU-H71 treatment led to a marked inhibition of other signaling trajectories pivotal for malignant cell survival leading to increased efficacy of chemotherapeutic agents potentially through induction of apoptosis (Bulatowicz and Wood, 2022).

Significantly, the PI3K/Akt pathway is a principal signaling cascade activated by IGF1R. Following IGF1R activation, PI3K is recruited and then activated, which in turn activates Akt. Of note, Akt promotes cell proliferation and survival through stimulating protein synthesis and inhibition of apoptotic pathways. PU-H71’s inhibition of IGF1R was reported to decrease Akt activation, thereby inducing apoptosis and inhibiting malignant cell proliferation (Gao et al., 2019).

Similar to the PI3K/Akt pathway, the Ras/Raf/MEK/ERK pathway was shown to be inhibited after IGF1R signaling suppression by PU-H71 leading to reduced cell proliferation and induction of apoptosis (Caldas-Lopes et al., 2009).

### 7.14 Cyclin-dependent kinase 4 (CDK4)

Cyclin Dependent Kinase 4 (CDK4) is defined as a serine/threonine kinase that plays a pivotal role in cell cycle progression. Initially, it complexes with cyclin D and phosphorylates the retinoblastoma protein (Rb) inducing the release of E2F transcription factors that in turn activate the expression of the genes required for the G1 to S phase transition (Pandey et al., 2019). Conversely, PU-H71 was reported to suppress CDK4, this effect could be mediated through either promoting degradation of HSP90 client proteins, including CDK4 (Serwetnyk and Blagg, 2021) or downregulating CDK4 (Knudsen and Witkiewicz, 2017).

A previous *in-vitro* study on breast cancer cells displayed that, PU-H71 attenuated CDK4 and induced G1 phase cell cycle arrest. Notably, PU-H71 exposure induced a profound suppression of the CDK4/cyclin D/Rb pathway through reducing protein expression of CDK4 and cyclin D1 with a subsequent decline in Rb phosphorylation (Pimienta et al., 2011). Additionally, PU-H71 demonstrated a prominent effect on other HSP90 client proteins central to abnormal cell proliferation, survival, and drug resistance, including epidermal growth factor receptor (EGFR), mammalian target of rapamycin (mTOR) and human epidermal growth factor receptor 2 (HER2) (Trendowski, 2015).

### 7.15 Interferon (INF)

The binding of PU-H71 to HSP90 will lead to the disruption of interferon (IFN) signaling. PU-H71 treatment reduces the release of IFNγ from activated T cells (Lisi et al., 2013). IFNγ is a key cytokine that mediates inflammatory and anti-viral responses. PU-H71 inhibits the activation of astrocytes by lipopolysaccharide and IFNγ, as measured by reduced nitric oxide synthase 2 (NOS2) expression and nitrite production where Activated astrocytes secrete inflammatory mediators like nitric oxide (Lisi et al., 2013).

The exact molecular mechanisms by which PU-H71 modulates interferon signaling are not fully elucidated. However, it likely involves the destabilization of key signaling proteins in the IFN pathway, such as JAK and STATs (Koppikar et al., 2012; Mbofung et al., 2017; Kuykendall et al., 2020). Degradation of these proteins by the proteasome upon HSP90 inhibition would impair IFN-induced gene transcription and the inflammatory response.

### 7.16 Janus kinase 2/signal transducers and activators of transcription 3 (JAK2/STAT3)

PU-H71 has shown promising antineoplastic effects in preclinical models of malignancies driven by aberrant JAK/STAT signaling (Kucine et al., 2015). PU-H71 can directly bind to oncogenic JAK2 mutants, such as JAK2V617F, which are common driver mutations in myeloproliferative neoplasms (MPNs) (Jego et al., 2019). This binding leads to the proteasomal degradation of mutant JAK2, abrogating its constitutive activation.

By binding to HSP90, PU-H71 disrupts the chaperone complex that maintains JAK2 in an active conformation. This results in a dose-dependent inhibition of JAK2 kinase activity and reduced phosphorylation of its downstream substrates (Rampal et al., 2014).

JAK2-mediated STAT3/STAT5 phosphorylation is a rate-limiting step in the activation of such oncogenic signaling cascade (Halim et al., 2020). Yet, STAT3/STAT5 phosphorylation was mitigated following PU-H71 exposure, which consequently prevented their dimerization, nuclear translocation, and transcriptional activity (Niu et al., 2021).

### 7.17 Human epidermal growth factor receptor 2 (HER2)

HER2 is a receptor tyrosine kinase that plays a crucial role in regulating the rate of cellular proliferation as well as cell survival. HER2 upregulation was reported to induce a remarkable increase in the rate of cell proliferation alongside apoptosis inhibition, contributing to the pathogenesis as well as the development of an array of malignancies, chiefly breast cancer (Hsu and Hung, 2016). Of note, approximately 20%–30% of diagnosed breast cancer cases demonstrated HER2 overexpression. Herein, HER2-positive tumors significantly demonstrated more aggressive behavior and poor prognosis compared to HER2-negative tumors (Cheng, 2024). Moreover, HER2 overexpression was documented in other malignancies (e.g., gastroesophageal adenocarcinomas, ovarian cancers, and endometrial cancers) (Iqbal and Iqbal, 2014).

HER2 activation involves its dimerization with other members of the ErbB family of receptors followed by provocation of downstream signaling axes, particularly, the PI3K/Akt and MAPK pathways (Elster et al., 2015) promoting cell proliferation, survival, and metastasis, highlighting HER2 as a significant druggable molecular target in our fight against cancer.

Conspicuously, HSP90 inhibition following PU-H71 treatment was shown to disrupt the chaperone’s interaction with HER2 stimulating its degradation and then reducing its levels in cancer cells (Li and Luo, 2023). Furthermore, PU-H71-mediated downregulation of HER2 expression was stated. Additionally, PU-H71 exposure displayed a significant impact on key oncogenic cascades indispensable for cancer cell survival and proliferation namely, ERK1/2 and Akt (Kale et al., 2020). The observed inhibition of these signaling cascades consequent to PU-H71 treatment could potentially induce significant cytotoxic effects in different molecular types of breast cancer, among which triple-negative breast cancer (TNBC) is the most important owing to the reported resistance to conventional therapies. This multimodal approach suggested that PU-H71 could be a promising candidate for the treatment of HER2-positive and other aggressive cancer types.

### 7.18 Epidermal growth factor receptor (EGFR)

EGFR is a key receptor tyrosine kinase, that belongs to the ErbB family that plays a pivotal role in regulating cellular proliferation as well as survival. EGFR is mostly overexpressed in a range of neoplasms (e.g., lung, brain and neck cancers) (Roskoski, 2014). Therefore, EGFR represents a potential druggable target for the development of novel targeted cancer therapies.

EGFR activation results in subsequent activation of other downstream cascades including PI3K/Akt pathway, Ras/Raf/MEK/ERK pathway, and Phospholipase C γ1 (PLCγ1)/PKC pathway (Martinelli et al., 2017; Salama et al., 2019; Guerra et al., 2021). Therefore, HSP90 inhibition by PU-H71 resulted in EGFR destabilization as well as degradation suppressing the activation of downstream cascades. This was shown to increase the sensitivity of cancer cells to conventional anti-cancer agents, especially, malignant cells harboring mutant forms of EGFR, which are often dependent on HSP90 for their stability (Lee et al., 2016).

### 7.19 Mammalian target of rapamycin (mTOR)

As previously stated, mTOR represents a key serine/threonine protein kinase central to regulating cell metabolism, growth, and survival in response to various environmental cues such as growth factors, nutrients, energy levels, and stress signals (Wu and Storey, 2021). In addition, The mTOR pathway was defined as a chief oncogenic pathway that contributed significantly to the pathogenesis of an array of malignancies as it was aberrantly hyperactivated by mutational activation of its upstream regulators (Popova and Jücker, 2021).

Notably, mTOR forms two distinct complexes, mTOR complex 1 (mTORC1) and mTOR complex 2 (mTORC2) both are key regulators of principal cellular processes. Significantly, mTORC1 is the primary regulator of protein synthesis, lipogenesis, and autophagy, while mTORC2 plays a remarkable role in regulating cell survival and cytoskeleton organization (Szwed et al., 2021). In cancer cells, constitutive activation of the mTOR pathway promotes several hallmarks of cancer, including increased protein synthesis, lipid metabolism, and inhibition of autophagy, which are vital for rapid cell growth and proliferation. Dysregulation of mTOR signaling has been implicated in various types of cancer, including lung, breast, liver, renal, pancreatic, and prostate cancers. PU-H71 was found to disrupt the mTOR pathway, leading to decreased protein synthesis and enhanced apoptosis in cancer cells (Giulino-Roth et al., 2017). This mechanism contributes to the therapeutic efficacy of PU-H71 in targeting mTOR-driven cancers.

PU-H71 exerts its effects through a range of molecular mechanisms targeting various pathways. It downregulates Ras, Raf-1, and ERK in MAPK signaling, reducing cell proliferation, enhancing radiosensitivity, and inducing G2/M phase arrest by disrupting cyclins and CDKs. PU-H71 inhibits Tyk2, impacting JAK/STAT, MAPK, and PI3K/Akt pathways to induce anti-proliferative and pro-apoptotic effects. It also induces PKC degradation by preventing HSP90 chaperoning. By inhibiting ATP production, PU-H71 disrupts glycolytic and oxidative metabolism. It activates mitochondrial apoptosis pathways, downregulating Bcl-2 and upregulating Bax, and targets ER HSP90 to destabilize client proteins. The drug affects HGFR by disrupting its stability and function, thereby reducing tumor growth. PU-H71 also inhibits Akt and IGF1R, affecting survival pathways and promoting apoptosis. It reduces inflammatory responses by decreasing IFNγ release and inhibits JAK2/STAT3 signaling. Finally, it targets HER2 and EGFR, reducing their activation and downstream signaling, thus inhibiting cell proliferation and promoting apoptosis.

## 8 Impact of PU-H71 on cancer

### 8.1 Impact on triple-negative breast cancer

Triple-negative breast cancer (TNBC) accounts for about 12% of all breast tumors and is more prevalent in premenopausal women of African and African-American heritage (Howard and Olopade, 2021). TNBCs are defined by their low degree of differentiation, and a significant majority of them are classified within the primary subtype of breast cancers based on gene manifestation analysis (Cserni et al., 2021; Fusco et al., 2022). In breast cancer, there is the existence of the molecular chaperone protein identified as HSP90. HSP90 plays a critical role in maintaining a substantial reservoir of active and properly folded oncoproteins. It has a distinct affinity for its activated form, serving as a safeguarding “biochemical buffer” that shields cancer-promoting oncogenes (Kunachowicz et al., 2024). TNBCs are distinguished by the absence of estrogen, progesterone, and HER2 receptor expression. Because there are no specific targets and targeted therapies available, as well as the diverse molecular makeup of TNBCs, the current treatment guidelines primarily rely on conventional chemotherapy. While this approach proves effective for certain patients, it leads to early relapse in a substantial portion of instances and does not offer a cure for individuals with metastatic TNBC (Medina et al., 2020; Vagia et al., 2020). The HSP90 inhibitor PU-H71 has demonstrated remarkable efficacy against these tumors. This agent has been shown to produce forcible and enduring anti-tumor impacts in TNBC xenografts, counting instances of full reaction and tumor reversion, free from inducing harm to the host (Anwar et al., 2020). Importantly, TNBC tumors have displayed continued responsiveness to PU-H71 retreatment for multiple cycles, extending over more than 5 months, with no signs of resistance or toxicity. Both in laboratory experiments (*in vitro*) and in living organisms (*in vivo*). PU-H71 has proven highly effective in consistently reducing and deactivating these proteins. It has been identified several mechanisms contributing to its anti-proliferative effects, including the suppression of factors in the Ras/Raf/MAPK pathway and the triggering of apoptosis by breaking down activated Akt and Bcl-xL. Additionally, PU-H71 inhibits various signaling pathways such as NF-κB, Akt, ERK2, Tyk2, and PKC, which collectively decrease the influential domination of TNBC (Caldas-Lopes et al., 2009; Ozgur and Tutar, 2014; He and Hu, 2018; Scott, 2018).

PU-H71’s cytotoxicity was evaluated in TNBC cell lines MDA-MB-468, MDA-MB-231, and HCC-1806 employing a test that measures ATP degrees (Caldas-Lopes et al., 2009). It had been noted that PU-H71 effectively prevented the expansion of these cells at concentrations known to bind to HSP90, indicating a correlation between its anti-tumor activity and its interaction with HSP90 (Chiosis et al., 2008). In an experiment conducted over a 48-h incubation period using an intensification of PU-H71 that was 5- to 10-fold more than its IC50 for expansion prevention (1 M), PU-H71 exhibited notable cytotoxicity (Anwar et al., 2020). Using PU-H71 as a data collection method offers preclinical evidence that achieving a comprehensive response is possible across molecularly diverse TNBC tumors using a single agent, an HSP90 inhibitor. The data collected with PU-H71 suggests that the variability in the effectiveness of other HSP90 inhibitors in TNBC tumors is not primarily due to a decrease in the number of HSP90-present tumors. Instead, it suggests that differences in their effectiveness may be attributed to factors unrelated to the target (HSP90), which remain undisturbed. In contrast to approaches that exclusively target specific proteins or signaling pathways within TNBC cells, PU-H71’s exceptional efficacy can be linked to its capacity to suppress HSP90, whether in laboratory settings (*in vitro*) or within living organisms (*in vivo*). HSP90 is a protein that forms complexes with numerous proteins pivotal for the cancerous characteristics of TNBC. PU-H71 induces concurrent instability and decrease of several crucial TNBC oncoproteins, a lot of which contribute to processes such as cell proliferation, anti-apoptotic capabilities, and metastasis.

### 8.2 Impact as cancer cell sensitizer for carbon-ion beam therapy

Carbon-ion radiotherapy (CIRT) has shown its efficacy in treating a range of tumor types, including lung cancer, prostate cancer, and bone/soft tissue cancer (Kamada et al., 2015). However, there is ongoing potential for further progress in the field of CIRT, especially in terms of effectively managing radioresistant tumors while minimizing adverse effects on healthy tissues. Radiosensitizers play a crucial role in enhancing radiotherapy by making tumor cells more responsive to radiation, enabling the achievement of similar therapeutic effects with lower radiation doses (Xie et al., 2019). While there is a wealth of research on radiosensitizers for X-ray-based therapies, there has been relatively limited investigation into radiosensitizers specifically designed for use with carbon-ion beams (Yanagi et al., 2009).

The prospect of targeting HSP90 for cancer treatment holds significant potential owing to its elevated expression in cancer cells in contrast to normal cells, and cancer cells exhibit a greater dependence on HSP90 for their survival (Jhaveri et al., 2012). Numerous inhibitors of HSP90 have been formulated and have demonstrated promising treatment efficacy; however, their therapeutic potential has been constrained due to associated toxicity. In several preclinical investigations, PU-H71 has demonstrated therapeutic efficacy in various preclinical models and is presently undergoing clinical trials. While CIRT has been effective in controlling tumors, there remains an opportunity for enhancement, particularly in terms of achieving tumor-specific radiosensitization (Li et al., 2016; Sokol and Durante, 2023).

There are currently two phase I clinical trials in progress, involving patients with solid tumors or lymphoma, So there is growing interest in PU-H71 as a promising novel drug (Li et al., 2016). PU-H71, regarded as one of the strong potential HSP90 inhibitors, is a resultant of PU-3 that has been created to possess both dispersibility and targeted binding affinity to the ATP-binding sites of HSP90 (Li et al., 2016). HSP90 operates through a sophisticated cycle regulation by ATP combination and dissociation. PU-H71 inhibits the function of HSP90 by preventing ATP from binding to it (Jhaveri et al., 2012). Inhibiting HSP90 represents an appealing approach to combined therapy, as there have been numerous reports indicating that HSP90 inhibitors can induce significant and lethal damage to tumor cells when used in binding with X-ray radiation therapy (Segawa et al., 2014). There have been only a limited number of studies examining the enhancing sensitivity of HSP90 inhibitors on high-LET C-ions, and the results of these studies remain a topic of debate and discussion (Musha et al., 2012). While PU-H71 when examined in isolation, there were no signs of toxicity to LM8 cells, it was demonstrated that after 24 h of incubation, PU-H71 significantly heightened the sensitivity of LM8 cells to both X-ray and C-ion exposure (Li et al., 2016). Due to the potent cell-killing capabilities of C-ions, making tumors more responsive to high-LET radiation, including C-ion therapy, through combination with other treatments, such as anti-cancer medications, can be particularly challenging (Li et al., 2016). Achieving successful combination therapy can indeed be complex. The potency of the standard treatment in a combination therapy approach should ideally surpass the effectiveness of each therapy component. Additionally, the side effects of the combined treatment must be less severe than the cumulative side effects regarding every specific therapy (Schirrmacher, 2019). Consequently, optimized doses for combinatorial treatment often should be administered at lower levels than the individual treatment doses to strike a balance between maximizing therapeutic benefit and minimizing adverse effects (Reece et al., 2007). PU-H71 has demonstrated the ability to sensitize LM8 cells at a dosage that doesn't disrupt cellular homeostasis. This suggests that PU-H71 holds noteworthy radiosensitizing qualities for CIRT (Li et al., 2015).

The potentially lethal impact of radiation on cells is primarily attributed to the induction of DNA double-strand breaks (DSBs) (Mladenov et al., 2013), HSP90 client proteins are prevalent in numerous DSB-associated proteins. Consequently, the focus was on exploring protein expression in the context of DSB repair and paying special attention to RAD51 and Ku70 levels (Pennisi et al., 2015; Khurana et al., 2016). These proteins play crucial roles in the primary cellular repair pathways, namely, homologous recombination and non-homologous end joining. Treatment with PU-H71 alone caused a reduction in RAD51 expression in LM8 cells (Ren et al., 2014). This suggests that PU-H71’s ability to enhance the sensitivity of C-ion therapy may also involve the non-homologous end-joining DSB repair pathway. Nevertheless, further investigation is necessary to fully elucidate the exact mechanism responsible for PU-H71’s radiosensitizing effect.

### 8.3 Impact on myeloma

In preclinical research, several HSP90 inhibitors have demonstrated effectiveness against myeloma, and at least three of these drugs have proceeded to Phase I/II clinical trials for the treatment of relapsed/refractory myeloma (Cavenagh et al., 2017). Preclinical studies utilizing PU-H71 in the treatment of diffuse large B-cell lymphoma and triple-negative breast cancer have indicated significant efficacy while causing minimal hematological or non-hematopoietic toxicity (Usmani et al., 2010). It is worth noting that the effectiveness of PU-H71 in treating multiple myeloma has not been thoroughly studied. However, in both IL-6 independent cell lines and IL-6 reliant cell line INA-6, PU-H71 has demonstrated comparable anti-myeloma efficacy (Usmani et al., 2010). Moreover, PU-H71 has exhibited *in vitro* synergy with other commonly used anti-myeloma medications. Additionally, PU-H71 has been shown to activate the UPR and induce apoptosis through the caspase-dependent pathway (Usmani et al., 2010). Over the past decade, there has been considerable research into the ER HSP90 family member known as gp96, or HSP90B1, also referred to as grp94. This research has been driven by gp96’s role in chaperoning antigenic peptides, enabling their presentation in the antigen-cross-presentation pathway (Marzec et al., 2012; Duan et al., 2021). Immunizations utilizing pure tumor-derived gp96-peptide complexes have demonstrated promising preclinical and clinical outcomes in the treatment of multiple myeloma (Qian et al., 2009). To assess the *in vitro* effectiveness of anti-myeloma drugs when gp96 was absent, researchers established a stable gp96 knockdown human myeloma cell line. The results aligned with expectations: bortezomib, known for disturbing protein balance, rendered gp96 knockdown cells more susceptible (Hua et al., 2013). Additionally, gp96 knockdown cells displayed heightened vulnerability to thalidomide, whose mechanism of action remains unidentified, as well as increased cell death induced by genotoxic drugs like melphalan and doxorubicin (Bagratuni et al., 2010; Hua et al., 2013). These findings imply that gp96 may play a role in reducing the efficacy of these anti-myeloma medications, and its absence amplified the sensitivity of myeloma cells to these agents (Usmani et al., 2010). However, the absence of gp96 had a significant negative impact on the anti-myeloma activity of PU-H71. This information indicates that HSP90 inhibitors affect not only cytosolic HSP90 but also gp96 in the ER, contributing to their acute cytotoxic effects (Hua et al., 2013). The novel purine-based HSP90 inhibitor, PU-H71, displayed *in vitro* anti-myeloma efficacy as well. These findings highlight that HSP90 inhibitors, including PU-H71, target both cytosolic HSP90 and the ER-resident HSP gp96 to exert their anti-myeloma effects. It is imperative to initiate a phase I clinical trial to assess the therapeutic efficacy of PU-H71 in individuals with refractory myeloma.

### 8.4 Effect on B-Cell lymphomas

The function of HSP90 and the impact of HSP90 inhibitors in the most popular kind of lymphoid malignancies, such as diffuse large B-cell lymphomas (DLBCL) that come from germinal center B-cells, remain largely unexplored and not well understood (Klein and Dalla-Favera, 2008). Bcl6, which is the most common oncogenic protein in DLBCL, has been identified as a client protein of HSP90 based on observations of how DLBCL cells respond to HSP90 inhibitors (Cerchietti et al., 2009; Calvo-Vidal et al., 2021). The interaction between HSP90 and Bcl6 influences the biology of normal germinal center B-cells as well as the pathophysiology of DLBCL to varying degrees (Ci et al., 2008). The functional and physical connection between HSP90 and Bcl6 may, in part, illustrate that treatment by directly controlling both Bcl6 mRNA and protein (Cerchietti et al., 2010). It is suggested that both mechanisms contribute to maintaining stable Bcl6 levels, although it is challenging to determine the precise extent to which HSP90 influences Bcl6 mRNA stability versus Bcl6 protein stability.

To establish germinal centers, where B-cells undergo affinity maturation, normal B-cells must undergo an upregulation process. HSP90 may play a role in supporting B-cell development by assisting in the accumulation of Bcl6 within germinal center B-cells, especially considering its localization in the nuclei of healthy Centro blasts (Hatzi and Melnick, 2014). These findings suggest that BCL6, which is part of a larger gene family subject to post-transcriptional regulation, could indeed function as a gene responsive to stress conditions (Cerchietti et al., 2009; Fernando et al., 2019; Liu et al., 2022). The fact that Bcl6 is a key target of HSP90 inhibitors in DLBCL aligns with the idea that it is essential for the survival of a subgroup of DLBCLs. This concept gains support from studies involving PU-H71, which showed that a version of Bcl6 resistant to degradation was enough to protect DLBCL cells (Culjkovic-Kraljacic et al., 2016). Studies like these emphasize the importance of discovering drugs that can enhance the effectiveness of conventional anti-lymphoma therapies while reducing their toxicity. It is noteworthy that HSP90 inhibitors have the propensity to accumulate preferentially within lymphomas for an extended period compared to normal tissues. In this regard, PU-H71 stands out as an exceptionally effective and fine-tolerated anti-lymphoma drug *in vivo*. Furthermore, finding that PU-H71 can play a role in the depression of Bcl6 target genes, that cause DNA damage checkpoints, raises the possibility that such medications could increase the cytotoxic activity of chemotherapy drugs. This effect was observed in various lymphoid cell lines, including DLBCLs (Zhao and Houry, 2005; Shinozaki et al., 2006).

### 8.5 Impact on human hepatocellular carcinoma

There has been a suggestion that HSP90 might be involved in the progression of hepatocellular carcinoma (HCC), potentially contributing to the hepatocarcinogenesis process (Mohammed et al., 2023a). Several factors that promote hepatocarcinogenesis, such as hepatocyte growth factor receptor, protein kinase B, IGF1R, cyclin-dependent kinase 4, and v-raf-1 murine leukemia viral oncogene homolog 1 exhibit abnormal expression and increased activity when influenced by HSP90. In general, HSP90 regulates the stability and activity of proteins represented as “client proteins,” (Saber et al., 2020; El-Kashef et al., 2022; Hasan Khudhair et al., 2022; Shaaban et al., 2022; Mohammed et al., 2023a; Mohammed et al., 2023b) and this is how it impacts protein homeostasis. Its activity is driven by adenosine triphosphate and involves dynamic interactions with co-chaperones (Workman et al., 2007; Pearl et al., 2008). The development of HCC is believed to be influenced by the upregulation of HSP90 in conjunction with increased CDK4 activity (Pascale et al., 2005). HCC typically leads to liver damage (Abdel-Ghany et al., 2015; Saber et al., 2018; Abdelhamid et al., 2022). Considering that geldanamycin analogs, which are HSP90 inhibitors, often cause dose-limiting hepatotoxicity, using HSP90 inhibition with these drugs may not be a suitable treatment option for this type of cancer (Pearl et al., 2008). Novel HSP90 inhibitors that do not contain the benzoquinone component found in geldanamycin, which is believed to contribute to hepatotoxicity, may offer potential prospects for HSP90 inhibition in the therapy of HCC in the future (Pearl et al., 2008). HCC is a major global health concern, with over 500,000 new cases diagnosed annually. It ranks as the sixth most common cancer worldwide (Abd El-Fattah et al., 2022; Nasr et al., 2023; Saber et al., 2023). Its incidence is on the rise globally (Nasr et al., 2023), and notably, it is the fastest-growing reason for cancer-related deaths among men in the United States (Bruix et al., 2004; El–Serag and Rudolph, 2007). Hepatitis B virus (HBV) and hepatitis C virus (HCV) infections are the primary factors contributing to the development of HCC. However, almost any condition that results in cirrhosis, with alcoholism being a prominent example, can potentially lead to the development of hepatocarcinogenesis (Farazi and DePinho, 2006; Abdelhamid et al., 2021).

The prognosis for HCC remains unfavorable because it is often diagnosed at an advanced stage, and tumor recurrence is relatively common (Bruix et al., 2004). Worrisome increases in HCV infection rates are raising concerns about the possibility of an impending HCC pandemic. This underscores the critical and immediate need for effective treatments to address this growing public health issue (El–Serag and Rudolph, 2007). Inhibiting HSP90 simultaneously reduced various factors that promote hepatocarcinogenesis, suppressed the growth of HCC tumors, and induced apoptosis (Nouri-Vaskeh et al., 2020). The use of non-quinone compounds as HSP90 inhibitors appears to be a promising treatment strategy for HCC. In animal studies, the non-quinone HSP90 inhibitor, PU-H71, specifically accumulated in tumors, effectively inhibited the growth of HCC tumors, and was well-tolerated, suggesting a low risk of severe liver toxicity (Breinig et al., 2009). In in vivo experiments, the non-quinone HSP90 inhibitor PU-H71 demonstrated no adverse effects on liver function, and no toxicity was observed (Speranza et al., 2018). This finding underscores the growing evidence that non-quinone HSP90 inhibitors are well-tolerated in living organisms (Rodina et al., 2007; Chandarlapaty et al., 2008; Eccles et al., 2008). The quick removal of PU-H71 from the liver, combined with its effective retention in tumors, improves the advantageous therapeutic characteristics of non-quinone HSP90 inhibitors for treating HCC. Additionally, the fact that PU-H71 treatment did not substantially affect ERK, AKT, or CDK4 in mouse livers further supports this conclusion (Breinig et al., 2009). These findings build upon prior research involving a different non-quinone HSP90 inhibitor, emphasizing the potential advantages of such inhibitors for HCC therapy (Chandarlapaty et al., 2008).

### 8.6 Impact on glial cells

Geldanamycin (GA) and radicicol are natural compounds known for their ability to inhibit the N-terminal ATPase activity of HSP90. HSP90 primarily acts as a chaperone to oversee a set of proteins known as “client proteins” during the final stages of their maturation and folding, especially as they near their native, functional configuration. HSP90 is heavily involved in various signal transduction processes (Barginear et al., 2008). When compounds like radicicol inhibit the ATPase activity of HSP90, client proteins detach from the chaperone (Roe et al., 1999). Among these client proteins, transcription factors like HSF-1 can become activated, while others may either lose their function or be marked for degradation by the proteasome. The removal of HSF-1 from HSP90 facilitates its movement into the nucleus, where it initiates the heat shock response (HSR). The use of HSP90 inhibitor drugs to stimulate the HSR pharmacologically can efficiently inhibit the pro-inflammatory activation of glial cells and enhance clinical outcomes in conditions such as experimental autoimmune encephalomyelitis (EAE) (Lisi et al., 2013; Seasons et al., 2024). The anti-inflammatory impacts of the HSR appear to be conveyed through a decrease in the activation of the NF-κB transcription factor (Schroeder et al., 2024). Ansamycins, including GA, are indeed potent HSP90 inhibitors. Nevertheless, their practical use in clinical settings is restricted due to notable toxicity concerns and stability challenges (Chiosis et al., 2004). PU-H71 is recognized as a potent HSP90 inhibitor, and datasets related to PU-H71 display a strong correlation in terms of HSP90 inhibition. This suggests that these drugs share a similar molecular method of action. The HSP90 inhibitor PU-H71 has demonstrated beneficial effects in the context of neuroinflammatory diseases (Kim et al., 2009).

PU-H71 was found to inhibit the activation of astrocytes when stimulated by the bacterial endotoxin lipopolysaccharide (LPS). However, it did not have an inhibitory effect on the activation of inflammatory cytokines. Similarly, PU-H71, like 17-AAG (another HSP90 inhibitor), had relatively mild anti-inflammatory influences on microglial initiation. Conversely to ansamycins (like GA), PU-H71 had a restricted impact on the progress of EAE disease. Its effects included a reduction in peripheral T-cell triggering and IFN release (Lisi et al., 2013). These data suggest that PU-H71 shares some anti-inflammatory roles with ansamycins. However, they also indicate significant disparities in their action mechanisms between ansamycins and PU-H71 regarding the initiation of glial cells, which may explain the lower performance of PU-H71 in EAE. Both 17-AAG and PU-H71 target blocking the ATPase activity of HSP-90 by binding to its N-terminal ATP/ADP pocket. Notably, the chemical structure of PU-H71 is entirely dissimilar from that of 17-AAG. PU-H71 has a simple formulation and lacks the benzoquinone moiety found in 17-AAG (Trendowski, 2015).

The primary difference between PU-H71 and 17-AAG lies in the absence of a benzoquinone group, potentially leading to distinct pharmacological effects. Interestingly, the presence or absence of DT-diaphorase appears to play a significant role in determining the varied response to HSP90 inhibition between the two compounds, possibly serving as a resistance mechanism (Kelland et al., 1999). Additionally, the silencing of the NAD (P) H dehydrogenase quinone 1 (NQO1) gene due to hypermethylation limits the effectiveness of 17-AAG in Hep3B cells, but this limitation does not apply to PU-H71 (Tada et al., 2005; Broekgaarden et al., 2015). Consequently, the anti-tumor response to novel HSP90 inhibitors is greatly enhanced by the NQO1 status (Hadley and Hendricks, 2014). The restricted *in vivo* effectiveness of PU-H71 may be attributed to differences in its selectivity for particular cells and its ability to penetrate the blood-brain barrier (BBB). It is important to highlight that, under normal circumstances, PU-H71 has a very restricted capacity to penetrate the CNS (Lisi et al., 2013). The comparatively modest inhibitory impact of the drug noticed *in vitro*, coupled with the activation of glial cells using a combination of cytokines, may aid in understanding the drug’s restricted effects in MOG-induced EAE following treatment. Cytokines such as TNF, IFN, and IL-1, which are closely linked to neuroinflammatory processes, may play a substantial role in this particular context (Sosa and Forsthuber, 2011). Therefore, the use of a combination of these cytokines represents a more pertinent physiopathological stimulus compared to bacterial endotoxin when trying to replicate the conditions that glial cells encounter in neurodegenerative disorders such as multiple sclerosis (MS) within a living organism. In live animal experiments, drugs that exhibit substantial anti-inflammatory effects in laboratory settings and reduce the activity of NOS2 triggered by inflammatory stimuli like TII have been shown to alleviate the clinical symptoms of EAE, which serves as a model for MS (Lubina-Dąbrowska et al., 2017). LPS functions by binding to the Toll-like receptor 4 (TLR4) receptor, while cytokines interact with and activate various receptors through separate intracellular signaling pathways (Ciesielska et al., 2021; Youssef et al., 2021a; Youssef et al., 2021b; Afroz et al., 2022). The anti-inflammatory action of PU-H71 in glial cells appears to involve the inhibition of NF-κB activation, as suggested by *in vitro* studies using stably transfected C6 glioma cells. This mechanism may explain the observed effects in reducing nitrite levels in response to both LPS (LI) and a combination of cytokines (TII). PU-H71, when used at concentrations of 30 nM for LI-stimulated C6 cells and 50 nM for TII-stimulated C6 cells, was found to decrease NFκB promoter activity while simultaneously reducing the production of nitric oxide (NO) (Lisi et al., 2013).

### 8.7 Impact on myeloproliferative neoplasms

Myeloproliferative neoplasms (MPNs) are set of clonal hematological malignancies that encompass conditions such as polycythemia vera (PV), chronic myeloid leukemia (CML), primary myelofibrosis (PMF) and essential thrombocythemia (ET). These disorders involve the overproduction of certain blood cells in the bone marrow (Luque Paz et al., 2023; Dunn et al., 2024; Guo et al., 2024). Although it has been over three decades since the clonal origin of these conditions stemming from stem cells was established, the genetic basis of BCR-ABL-negative MPNs stayed enigmatic until multiple studies revealed a somatic activating mutation in the JAK2 kinase (JAK2V617F). This mutation is present in the overwhelming majority of PV patients and is observed in nearly 50% of patients with ET and PMF (James et al., 2005; Zhao et al., 2005). Follow-up investigations have uncovered somatic mutations in JAK2 exon 12 in instances of PV where the JAK2V617F mutation is absent. Furthermore, mutations in the thrombopoietin receptor, specifically MPLW515 L/K/A and MPLS505N, have been detected in a subset of patients with ET and PMF who do not possess the JAK2V617F mutation (Mario, 2007; Pietra et al., 2008). *In vitro*, the presence of JAK2/MPL mutations allows hematopoietic cells to proliferate even without the need for cytokines. This leads to increased activation of signaling pathways downstream of JAK2, including the STAT3/5, MAP kinase, and PI3K signaling pathways (Anand et al., 2011; Staerk and Constantinescu, 2012; Rao et al., 2019). Additionally, it is important to highlight that JAK2 or MPL mutations lead to a remarkably consistent pattern of myeloproliferation *in vivo*. This consistency is particularly notable in the context of conditions such as polycythemia (linked with JAK2V617F or JAK2 exon 12 mutations) and thrombocytosis/myelofibrosis (associated with JAK2V617F and MPLW515L mutations) (Wernig et al., 2006), This implies that the persistent activation of the JAK-STAT signaling pathway plays a crucial role in the progression of PV, ET, and PMF. PU-H71 demonstrates enhanced pharmacokinetic and pharmacodynamic characteristics, including its robust and sustained absorption by tumors. As a result, it leads to more efficient and enduring degradation of HSP90 client proteins (Jhaveri et al., 2020). Increasing PU-H71’s *in vivo* effectiveness doesn't appear to raise toxicity concerns. Extended treatment at effective doses has not shown significant hematopoietic or non-hematopoietic toxicities (Usmani et al., 2010; Levine et al., 2012).

Scientists used PU-H71 to assess HSP90 inhibition in JAK2-dependent cancers. PU-H71 demonstrated substantial anti-cancer effects in MPN cell lines, mouse models of MPN, and samples from MPN patients. PU-H71 treatment repressed cell proliferation in cells with JAK2/MPL mutations at doses related to JAK2 reduction and the suppression of downstream signaling pathways (Campbell and Green, 2006). Furthermore, giving PU-H71 to mice with JAK2V617F or MPLW515L mutations normalized blood parameters, reduced extramedullary hematopoiesis in both models, and decreased morbidity and mortality in the MPLW515L model, compared to mice receiving a control vehicle. Importantly, this therapeutic effect was achieved without causing hematopoietic or non-hematopoietic toxicities (Marubayashi et al., 2010). Additionally, PU-H71 treatment has been shown to inhibit abnormal erythrocytosis and megakaryopoiesis in the JAK2V617F and MPLW515L rodent models, while it does not impact normal erythrocytosis and megakaryopoiesis (Koppikar et al., 2010). These findings suggest that HSP90 inhibitor therapy with PU-H71 has a unique impact on the proliferation and signaling of the malignant clone. The specific effect of PU-H71 on JAK2/MPL mutant cells *in vivo* does not seem to be solely due to the mutant or activated JAK2’s increased reliance on the HSP90 chaperone complex (Marubayashi et al., 2010; Mullally, 2017).

Unlike previous experiments with a JAK2 inhibitor, PU-H71 treatment reduces the burden of mutant alleles in the MPLW515L murine MPN model. This strongly supports the idea of conducting clinical trials with PU-H71 for treating JAK2V617F/MPLW515L mutant MPN. In preclinical studies of PU-H71 and other HSP90 inhibitors, flow cytometric assays measuring JAK2 protein expression, phospho-STAT5 levels, and HSP70 induction could be utilized as pharmacodynamic assessments (Koppikar et al., 2010). PU-H71 and other HSP90 inhibitors affect numerous client proteins, likely contributing to their impact on myeloproliferation both *in vitro* and *in vivo*. However, evidence suggests that JAK2 is the primary target for HSP90 inhibitors in JAK2/MPL-mediated myeloproliferation (Weigert et al., 2012; Chakraborty et al., 2016; Hobbs et al., 2018). PU-H71 consistently resulted in JAK2 degradation and pathway inhibition *in vitro* and *in vivo* at comparable doses. Additionally, PU-H71 and two different JAK2 kinase inhibitors had a complementary, not synergistic, effect, implying a mutual mechanism of action in this situation (George et al., 2004). PU-H71 can serve as a valuable tool for identifying tumors depending on HSP90 chaperone proteins. This data could be integrated into genomic and proteomic databases to reveal new molecular targets across various human cancer types. The effectiveness of HSP90 inhibition by PU-H71 extends to a range of genetically diverse human cancers, highlighting the importance of conducting targeted drug trials with HSP90 inhibitors for treating MPNs and other malignancies (Gao et al., 2007; Hedvat et al., 2009).

## 9 The therapeutic potential of PU-H71 in non-tumor disorders

The reasoning behind the connection between protein aggregation and neurodegenerative conditions has been that blocking HSP90 leads to the activation of HSF-1, resulting in the production of HSP70, HSP40, and other chaperones, which in turn facilitate the disaggregation and breakdown of proteins. However, recent findings indicate that HSP90 has an added involvement in neurodegenerative processes. Specifically, HSP90 is involved in upholding the operational integrity of neuronal proteins that have an abnormal tendency, thereby permitting and sustaining the buildup of detrimental protein clumps (Waza et al., 2006).

Parkinson’s disease (PD), the most common neurodegenerative movement disorder, is marked by a complex interplay of pathological events (Westerlund et al., 2010). A substantial number of these events have been more recently associated with HSP90. Wang et al. have pointed out that Leucine-rich repeat kinase 2 (LRRK2), an enzyme whose altered forms already are found in both familial and sporadic cases of Parkinson’s disease, interacts with HSP90 *in vivo* to form a complex (Wang et al., 2008). The purine-based HSP90 inhibitor PU-H71 disrupted the interaction between HSP90 and LRRK2, resulting in the degradation of LRRK2 via the proteasome system. PU-H71 also decreased the neurotoxic effects induced by mutant LRRK2 and reversed the impairment in axon growth associated with the LRRK2 G2019S mutation in neurons (Wang et al., 2008). PARK6, a subtype of familial Parkinson’s disease inherited in an autosomal recessive fashion, is linked to a mutation that occurs in PTEN-induced kinase 1 (PINK1). PINK1 is a gene that codes for a potential mitochondrial serine/threonine kinase (Westerlund et al., 2010). The autosomal recessive inheritance pattern associated with this type of Parkinson’s disease indicates a strong connection between the disease’s development and the loss of PINK1 function. HSP90 always attaches to PINK1 to enhance its equilibrium. In cells exposed to the HSP90 inhibitor GM, the levels of PINK1 were significantly diminished through the ubiquitin-proteasome pathway (Moriwaki et al., 2008).

## 10 Conclusion

In this comprehensive review, we have explored the pivotal role of HSP90 as a key molecular player in maintaining cellular health and its intricate involvement in various disease contexts. We discussed how HSP90, in partnership with HSF-1, orchestrates stress responses, shedding light on its role in regulating the HSR. The therapeutic potential of PU-H71, a novel purine-based HSP90 inhibitor, has been a central focus of our investigation. We have elucidated its promise in addressing neurodegenerative disorders and its significant impact on triple-negative breast cancer, offering new avenues for cancer treatment. PU-H71’s ability to sensitize cancer cells for carbon-ion beam therapy suggests a novel approach to improving the efficacy of radiation therapy. Furthermore, the dual inhibition of HSP90 A and HSP90B1 by PU-H71 emerges as a potential breakthrough in myeloma treatment. Its capacity to induce apoptosis in B-cell lymphomas dependent on Bcl6 for survival underscores its relevance in hematologic cancers. Our exploration extends to HCC, where innovative chemotherapy approaches and PU-H71’s potential to inhibit the activation of glial cells hold promise for patients with hepatic and neuroinflammatory disorders. Finally, we emphasize PU-H71’s encouraging role in JAK2-dependent myeloproliferative neoplasms, highlighting its potential as a therapeutic avenue in hematology. Collectively, this review underscores the versatility of HSP90 and the immense therapeutic potential of PU-H71 in diverse disease scenarios, opening new doors to innovative treatments and improved patient outcomes in the realm of health and disease.

## 11 Authors’ perspectives and future considerations

From the author’s perspective, the multifaceted roles of HSP90 and the promising therapeutic potential of PU-H71 underscore the need for continued research and development in this field. Shedding more light on the underlying mechanisms of HSP90 and its inhibitors highlights them both as promising candidates for the development of novel targeted cancer therapies. Markedly, special attention should focus on optimizing precise drug delivery strategies to enhance the efficacy as well as selectivity of such novel therapeutic agents and to explore any potential combination therapies for maximum efficacy.

In addition, pondering the molecular pathways underlying PU-H71 actions is indispensable for tailoring novel therapeutic approaches to fulfilling specific patient needs. Further investigations on the long-term safety profiles of HSP90 inhibitors in addition to their potential for resistance mechanisms should be conducted to ensure their clinical success. The integration of pioneering technologies (e.g., precision medicine approaches and advanced imaging techniques) could potentially establish a platform for developing personalized as well as HSP90-targeted therapies.

## Glossary

17-AAG: 17-Allylamino-17-demethoxygeldanamycin
17-DMAG: 17-Dimethylaminoethylamino-17-demethoxygeldanamycin
Akt: Protein Kinase B
ATF4: Activating Transcription Factor 4
ATP: Adenosine Triphosphate
ATPase: Adenosine Triphosphatase
Bax: Bcl-2-Associated X Protein
Bcl-xL: B-Cell Lymphoma-extra Large
Bcl6: B-Cell Lymphoma 6
Bcl-2: B-Cell Lymphoma 2
BCoR: BCL6 Corepressor
C-ions: Carbon Ions
CIRT: Carbon-Ion Radiotherapy
CDK: Cyclin-Dependent Kinase
CHOP: C/EBP Homologous Protein
CK2: casein kinase II
DNA-PKcs: DNA-Dependent Protein Kinase Catalytic Subunit
DSB: DNA Double-Strand Break
DLBCL: Diffuse Large B-Cell Lymphoma
ER: Endoplasmic Reticulum
ERK: Extracellular Signal-Regulated Kinase
FoxO: Forkhead Box O
GRP78: Glucose-Regulated Protein 78
gp96: Glucose-Regulated Protein 94
HDAC: histone deacetylase
HSP27: Heat Shock Protein 27
HSP40: Heat Shock Protein 40
HSP60: Heat Shock Protein 60
HSP70: Heat Shock Protein 70
HSP72: Inducible Heat Shock Protein 70
HSP90: Heat Shock Protein 90
HSP90α: Heat Shock Protein 90 Alpha
HSP90β: Heat Shock Protein 90 Beta
HSP90B1: Heat Shock Protein 90 Beta Family Member 1
HSC70: Constitutive Heat Shock Protein 70
HSF-1: Heat Shock transcription Factor 1
HSR: Heat Shock Response
IC50: Half Maximal Inhibitory Concentration
IL-6: Interleukin 6
INF: Interferon
IGF1R: Insulin-Like Growth Factor 1 Receptor
JAK: Janus Kinase
LET: Linear Energy Transfer
MAPK: Mitogen-Activated Protein Kinase
mTORC: Mammalian Target of Rapamycin Complex
NF-κB: Nuclear Factor kappa-light-chain-enhancer of activated B cells
NOS2: Nitric Oxide Synthase 2
PKC: Protein Kinase C
PDK1: 3-Phosphoinositide-Dependent Protein Kinase-1
PI3K: Phosphoinositide 3-Kinase
PTMs: post-translational modifications
RAD51: RAD51 Recombinase
Raf: v-Raf-1 Murine Leukemia Viral Oncogene Homolog 1
Ras: Rat Sarcoma (a family of related proteins)
SMRT: Silencing Mediator for Retinoid and Thyroid Receptors
STAT: Signal Transducers and Activators of Transcription
TNBC: Triple-Negative Breast Cancer
Tyk2: Tyrosine Kinase 2
UPR: Unfolded Protein Response
VEGFR2: vascular endothelial growth factor receptor 2
XBP1: X-Box Binding Protein 1
